# Imaging Techniques: Essential Tools for the Study of SARS-CoV-2 Infection

**DOI:** 10.3389/fcimb.2022.794264

**Published:** 2022-07-22

**Authors:** Aurélie Deroubaix, Anna Kramvis

**Affiliations:** ^1^ Hepatitis Virus Diversity Research Unit, Department of Internal Medicine, School of Clinical Medicine, Faculty of Health Sciences, University of the Witwatersrand, Johannesburg, South Africa; ^2^ Life Sciences Imaging Facility, University of the Witwatersrand, Johannesburg, South Africa

**Keywords:** SARS-CoV-2, COVID-19, microscopy, diagnostic, functional studies, super-resolution, models, high content screening

## Abstract

The world has seen the emergence of a new virus in 2019, SARS-CoV-2, causing the COVID-19 pandemic and millions of deaths worldwide. Microscopy can be much more informative than conventional detection methods such as RT-PCR. This review aims to present the up-to-date microscopy observations in patients, the *in vitro* studies of the virus and viral proteins and their interaction with their host, discuss the microscopy techniques for detection and study of SARS-CoV-2, and summarize the reagents used for SARS-CoV-2 detection. From basic fluorescence microscopy to high resolution techniques and combined technologies, this article shows the power and the potential of microscopy techniques, especially in the field of virology.

## Introduction

At the end of 2019 a severe respiratory disease was noted in Wuhan, China, which was officially named coronavirus disease 2019 (COVID-19: CO for *Corona*, VI for *virus*, D for *disease*, 19 referring to *2019*, the year of its emergence) by the World Health Organization (WHO) on 11 February 2020 (WHO, 2020b). A month later, on the 11 March 2020, WHO declared COVID-19 a pandemic (WHO, 2020a), which continues to affect our world. Patients have a wide range of symptoms, from asymptomatic to mild to moderate respiratory symptoms and pneumonia and fulminant disease, which can lead to death in 2-5% of the cases (Petersen et al., 2020; Wu et al., 2020a). Symptoms vary and can include gastro-intestinal symptoms, multi-organ infections (Gu et al., 2005) and central nervous system damage (Ellul et al., 2020). By May 2022, in 223 countries, there have been more than 517 million confirmed cases of COVID-19 worldwide, including 6.3 million deaths, reported to the World Health Organization (WHO).

The causative agent of COVID-19 is a novel member of the family *Coronaviridae*, genus *Betacoronavirus* belonging to the subgenus *Sarbecovirus*, initially designated 2019-novel coronavirus (2019-nCoV) (Gorbalenya et al., 2020). It was named SARS-CoV-2 by the International Committee on Taxonomy of Viruses (ICTV) and belongs to the species Severe Acute Respiratory Syndrome-related coronavirus. Other members of the genus include HCoV-HKU1, HCoV-OC43, Middle East respiratory syndrome coronavirus (MERS-CoV), and the SARS-CoV, which infect humans. SARS-CoV-2 shares genetic similarities with these coronaviruses, especially the SARS-CoV from the 2003 outbreak (79% similarity) (Alanagreh et al., 2020; Rabaan et al., 2020).

**Figure 1:**
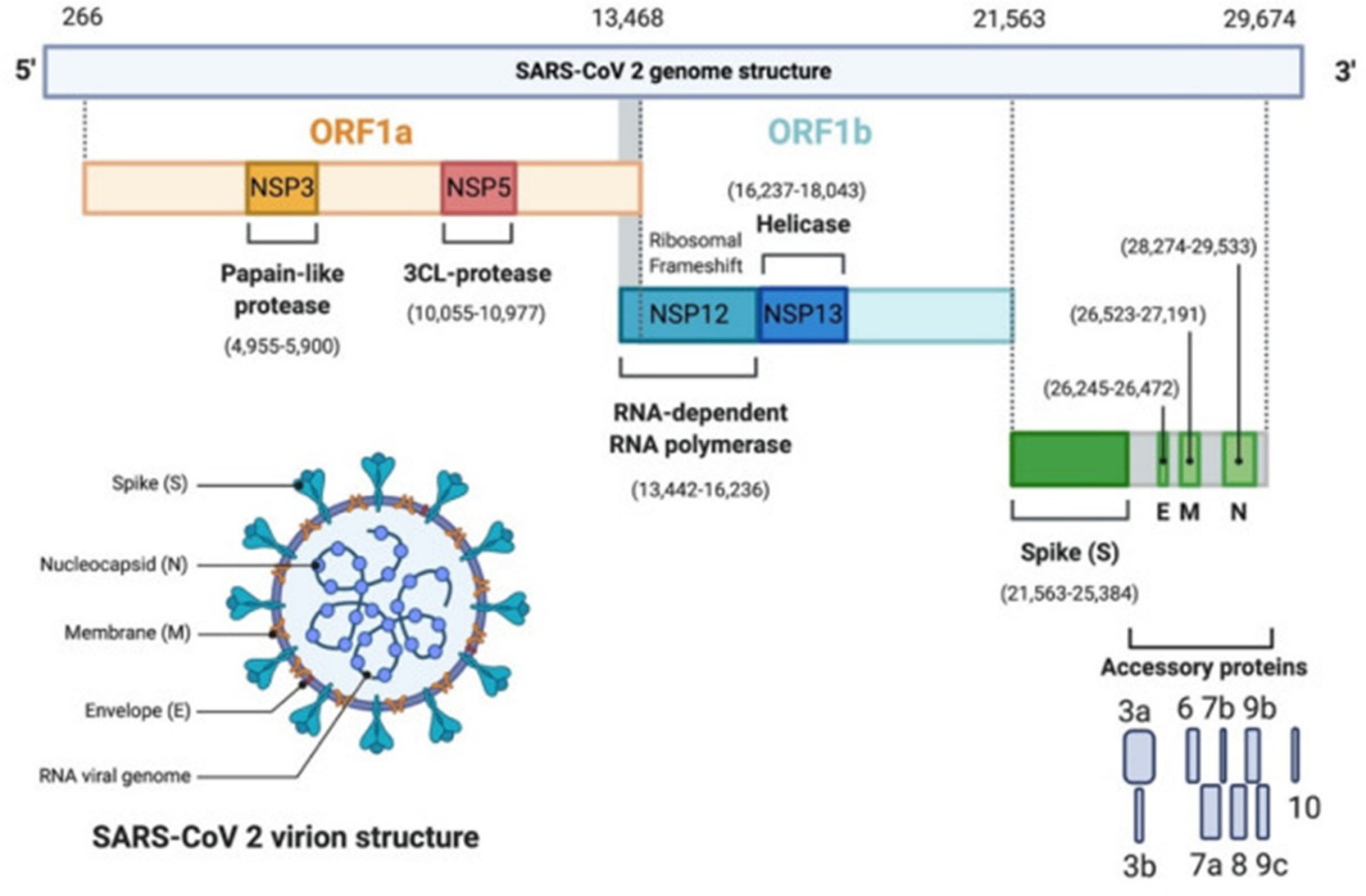
SARS-CoV-2 genomic organization. SARS-CoV-2 genome contains 2 ORFs, ORF1a (yellow) and ORF1b encoding 16 non-structural proteins (NSP1-16). NSP3 corresponds to the Papain-like protease, NSP5 the 3CL-protease, NSP12 the RNA-dependent RNA polymerase (RdRp), and NSP13 the helicase. It has 4 structural proteins: Spike (S), Envelope (E), Membrane (M) and Nucleocapsid (N) (green). S, E and M form the envelope, N associates with the viral genome. In grey, are the accessory proteins: 3a, 3b, 6, 7a, 7b, 8, 9b, 9c, 10. The viral genome is a 30 kb linear single-stranded RNA. Adapted with permission from Alanagreh et al. (Alanagreh et al., 2020).

Coronaviruses share the same structure and replication mechanism (Alanagreh et al., 2020) (**Figure 1**). They are single-stranded positive-sense RNA (+ssRNA) viruses. The genome contains at least 6 open reading frames (ORFs). ORF1a and b encode 16 non-structural proteins (NSP1-16). These NSPs are processed through the replication-transcription complex (RTC). For example, NSP3 and 5 encode proteases, NSP12 encodes the RNA-dependent RNA polymerase, NSP13 encodes a helicase. At least 4 other ORFs encode for 4 structural proteins: envelope glycoprotein spike (S), membrane (M) proteins, envelope (E) proteins and the nucleocapsid (N) proteins.

**Figure 2:**
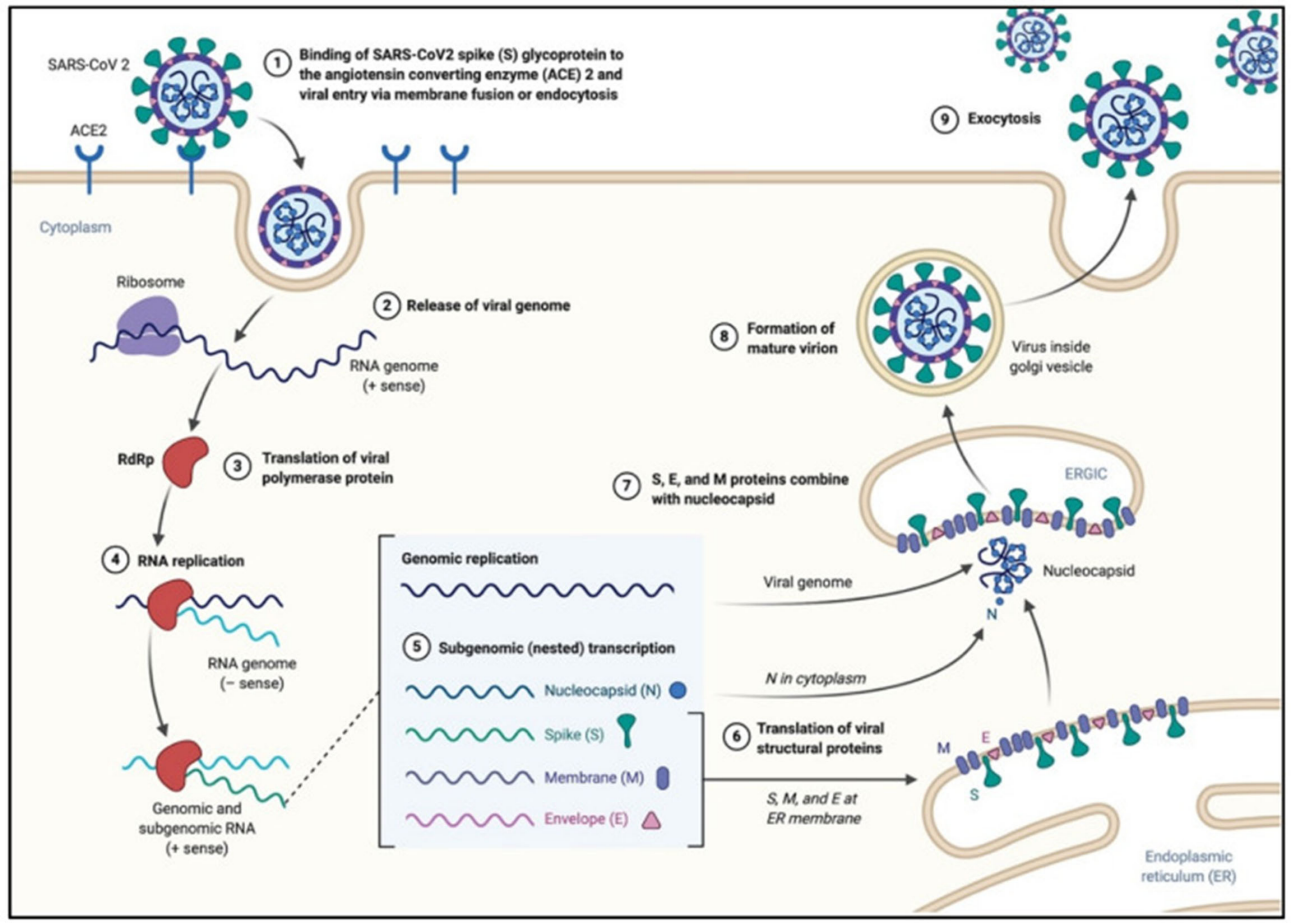
SARS-CoV-2 viral life cycle. The virus binds to the ACE2 receptor, proteolytic cleavage of the spike protein leads to the fusion of the viral envelope with the host membrane, leading to the formation of an endosome, and the release of the viral RNA. The viral polymerase (RNA dependent RNA polymerase RdRp) is translated. The (-)RNA is converted into (+)RNA and the proteins are translated. Non-structural proteins together with the RNA form the RNA-transcription complex. The genomic and subgenomic RNA are produced, and the structural proteins (spike, nucleocapsid, membrane and envelope are translated and new virions assemble at the endoplasmic reticulum). Adapted with permission from Alanagreh et al. (Alanagreh et al., 2020).

The viral spike protein binds to the cellular angiotensin-converting enzyme 2 (ACE2) receptor and enters the cell *via* an endosome. The viral RNA is released in the cytoplasm. ORFs 1a and 1b are translated into pp1a and pp1b polyproteins, which are cleaved by the proteases of the RTC. RNA replication occurs and produces negative RNA copies, which are used as templates for the production of (+) RNA genomes. Structural proteins are produced, nucleocapsids are assembled in the cytoplasm and budding occurs at the endoplasmic reticulum (ER)-Golgi intermediate compartments. Virions are then released *via* exocytosis (**Figure 2**).

While improved preventive measures, vaccines and treatments against COVID-19 (Jeong et al., 2020; Wu et al., 2020b) continue to be developed, it is essential that cutting edge technologies are utilized. Imaging techniques are essential tools in research, crucial for a better understanding of the host-pathogen relationship and pathogenesis, which can be useful in diagnosis and drug testing. A large selection of microscopy techniques is available, each with its strengths and limitations (**Table 1, Supplementary**).

The aim of the present review is to discuss classical and new microscopic techniques that have been used in order to:

- Detect the virus, describe the cellular, tissue and organ tropism of SARS-CoV-2
- Elucidate the molecular biology of SARS-CoV-2 and the host-pathogen interactions
- Understand the pathogenesis of the infection and its clinical consequences
- Develop diagnostic assays with high content screening
- Facilitate drug and vaccine development

This review also includes:

- A comparison of the microscopy techniques, their sensitivity and specificity
- The challenges encountered when using microscopy and the solutions to overcome them
- The challenges encountered for viral detection in different tissues especially autopsies
- The description of image analysis tools and the use of artificial intelligence
- The description of model systems for the study of SARS-CoV-2
- The list of new microscopy technologies for the study of SARS-CoV-2 and viruses in general.

This up-to-date literature review summarizes both the methods and the reagents available for the various imaging techniques, in order to provide an important resource for ongoing and future experimentation, necessary to combat this pandemic of unprecedented proportions.

## Multiple Tropisms of SARS-Cov-2: Use of Microscopy for the Detection of SARS-CoV-2 in Various Organs and Cells

The sudden emergence of an unknown virus, SARS-CoV-2, its contagiousness and the broad range of symptoms following infection required discovery of its organ and cellular targets. Detection in cells, tissues and organs can provide insights into its modes of transmission and pathogenicity.

**Figure 3:**
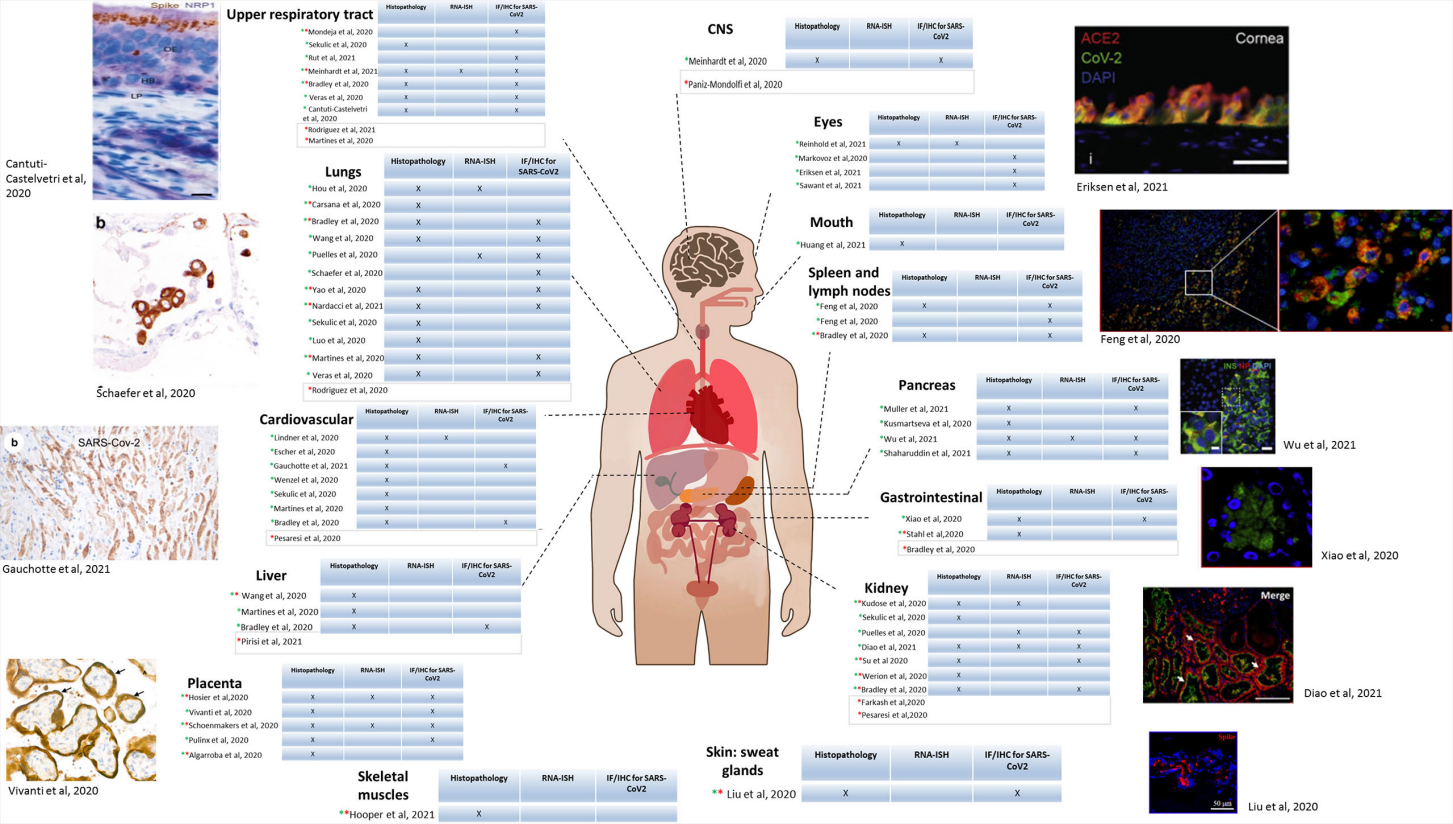
SARS-CoV-2 tropism in patients determined by microscopy. Diagram of the human body depicting each organ in which SARS-CoV-2 has been detected in biopsies from patients. For each organ, a table is included indicating the method used for detection. Column 1: histopathology; Column 2: RNA *in situ* immunohistochemistry (RNA-ISH),; Column 3: Immunofluorescence (IF) and/or Immunohistochemistry (IHC). Green star indicates light microscopy performed, and red star electron microscopy. For the studies having light microscopy done, an example of microscopic image is included. **Upper respiratory tract:** Apical olfactory epithelium (OE) of a COVID-19 patient; LP, lamina propria; HB, horizontal basal cells. Brown: spike protein; blue: NRP1 (Cantuti-Castelvetri et al., 2020) **Lungs:** IHC on lung sections showing SARS-CoV-2 positivity in pneumocytes. Co-staining showing co-localization of TTF-1 (brown)/SARS NC (red). (Schaefer et al., 2020) **Cardiovascular:** Staining of cardiomyocytes, NC protein. (ICC/X200). (Gauchotte et al., 2021) **Placenta:** Transplacental transmission of SARS-CoV-2 infection. Immunostaining for SARS-CoV-2 protein (X800) showing brown staining of peri-villous trophoblastic cells. (Vivanti et al., 2020) **Eyes:** Immunostaining of post-mortem human ocular surface tissue (limbal region). Green: SARS-CoV-2 spike protein, Red: ACE2 receptor. Blue: DAPI. (Eriksen et al., 2021) **Spleen and lymph nodes:** Immunofluorescence of spleen and lymph node tissue for SARS-CoV-2 NP (green); red: CD169 marker, blue: DAPI. Scale bar: 100 μM. (Feng et al., 2020a) **Pancreas:** Staining of pancreatic islets from COVID-19 patients. Red: SARS-CoV-2 NP. Green: insulin. (Diao et al., 2021) Scale bar: 5 µm. (Wu et al., 2021a) **Gastrointestinal tract:** Immunofluorescent staining of stomach tissues. Green: NP, blue: DAPI Scale bar: 20 μm. (Xiao et al., 2020) **Kidney:** kidney sections from COVID-19 post-mortem. Green: ACE2; Red: Spike protein; blue: Dapi. Arrows: positive tubules. Scale bars: 100 μm **Skin:** Immunofluorescence analysis in sweat glands infected by SARS-CoV-2 from deceased donors. Red: spike protein, blue: DAPI. Scale bar: 50 µm. (Liu et al., 2020a) Upper respiratory tract: (Bradley et al., 2020; Martines et al., 2020; Sekulic et al., 2020; Veras et al., 2020; Meinhardt et al., 2021; Mondeja et al., 2021; Rodriguez et al., 2021; Rut et al., 2021) Lungs: (Bradley et al., 2020; Cantuti-Castelvetri et al., 2020; Carsana et al., 2020; Hou et al., 2020; Luo et al., 2020; Martines et al., 2020; Puelles et al., 2020; Schaefer et al., 2020; Sekulic et al., 2020; Veras et al., 2020; Wang et al., 2020a; Yao et al., 2020; Nardacci et al., 2021; Rodriguez et al., 2021) Cardiovascular: (Bradley et al., 2020; Escher et al., 2020; Lindner et al., 2020; Martines et al., 2020; Pesaresi et al., 2020; Sekulic et al., 2020; Wenzel et al., 2020; Gauchotte et al., 2021) Liver: (Bradley et al., 2020; Martines et al., 2020; Wang et al., 2020d; Pirisi et al., 2021) Placenta: (Algarroba et al., 2020; Hosier et al., 2020; Pulinx et al., 2020; Vivanti et al., 2020; Schoenmakers et al., 2021) Skeletal muscles: (Hooper et al., 2021). CNS: (Paniz-Mondolfi et al., 2020; Meinhardt et al., 2021) Eyes: (Makovoz et al., 2020; Eriksen et al., 2021; Reinhold et al., 2021; Sawant et al., 2021) Mouth: (Huang et al., 2021) Spleen and lymph nodes: (Bradley et al., 2020; Feng et al., 2020a) Pancreas: (Kusmartseva et al., 2020; Muller et al., 2021; Shaharuddin et al., 2021; Wu et al., 2021a). Gastrointestinal: (Bradley et al., 2020; Stahl et al., 2020; Xiao et al., 2020). Kidney: (Kudose et al., 2020; Puelles et al., 2020; Sekulic et al., 2020; Diao et al., 2021)( (Bradley et al., 2020; Farkash et al., 2020; Pesaresi et al., 2020; Su et al., 2020; Werion et al., 2020). Skin, sweat glands: (Liu et al., 2020a).

Using imaging techniques (light and/or electron microscopy), SARS-CoV-2 and the consequences of the infection on cell morphology, together with immune cell infiltration, have been studied in several organs and tissues, including lungs (Tian et al., 2020; Yao et al., 2020), upper respiratory tract (nose, pharynx, larynx) but also heart (Lindner et al., 2020), kidneys, spleen and lymph nodes, liver, pancreas, mouth, gastrointestinal tract, skin, skeletal muscles, eyes, central nervous system (Nampoothiri, 2020; Paniz-Mondolfi et al., 2020) and the placenta (**Figure 3**). The frequency of infection of the different organs and tissues correlates with the concentration of ACE2 receptors (Trypsteen et al., 2020).

### Light Microscopy/Immunohistochemistry/Immunofluorescence

Biopsies were stained with routine histopathological techniques such as haematoxylin and eosin (H&E) and 3’diaminobenzidine (DAB), and could be analyzed with a simple light microscope. These techniques allowed histological analysis of patients’ tissues and labelling of infiltrated immune cells such as lymphocytes, monocytes, macrophages, intervillous fibrin depositions, analysis of epithelial cells and hyaline membranes.

In addition, fluorescent labelling was performed with antibodies specific to SARS-CoV-2 proteins (SARS-CoV-2 spike or nucleocapsid antibodies, with development of protocols for their detection and the detection of the viral RNA) (Carossino et al., 2020; Feng et al., 2020a; Lean et al., 2020; Sekulic et al., 2020) and host proteins (for example using markers for CD68 and CD3) (Bradley et al., 2020). Although the typical epifluorescence microscope is used daily in a routine laboratory, confocal microscopy was also used for viral detection (Hou et al., 2020; Nampoothiri, 2020; Werion et al., 2020; Xiao et al., 2020; Mondeja et al., 2021) bringing higher resolution and acquisition speed. In confocal microscopy, although the entire sample volume is illuminated, optical sectioning is possible due to the existence of a pinhole, which blocks the light coming from outside the focal plane. These results in the improvement of the resolution compared to an epifluorescent microscope. Confocal microscopy has become popular in the last decades, because it allows the acquisition of high quality images by getting images from focal planes, eliminating the out of focus background and thus increasing the resolution (Elliott, 2020). A novel technique in the 1980s, it is now accessible in low-income countries and can perform multiple analyses, as demonstrated for SARS-CoV-2.

### Challenges in IHC/Immunofluorescence

Many challenges have been identified (Von Stillfried and Boor, 2021). Positive and negative controls are not easy to obtain and most antibodies have not been validated by the manufacturer, with their specificity questioned (Baeck et al., 2020). Most of the antibodies cross-react between SARS-CoV and SARS-CoV-2, with unclear cross-reactivity with other viruses. There is no unique and established reference for histomorphological changes due to SARS-CoV-2, which shows identical changes to SARS-CoV. Heterogeneous results are observed in cells from the same organ. Additionally, some tissues, such as the placenta yield false-positive signals (Roberts et al., 2021).

The post-mortem detection of the virus is challenging, as it depends on different parameters, including the post-mortem interval, the type of organ, the viral load before death, the type of storage, the temperature/humidity, the burial process, the test material (swabs, cryopreserved tissues, fixed tissues, formalin-fixed paraffin-embedded tissues) (Von Stillfried and Boor, 2021). Also, possible autolysis and degradation may take place in the body.

### Electron Microscopy

Electron microscopy (EM) plays a role in viral identification/diagnosis with detection of viral morphology. For some, it is considered as a frontline method for rapid detection before any further investigation (Hazelton and Gelderblom, 2003). However, whether transmission EM (TEM) is a quick technique for detection is debatable and it is usually not used routinely as preparation is time consuming, it is not practical for a large number of samples, specific reagents are needed, and it is an expensive microscope that not every facility can afford or has the capacity to operate.

Scanning electron microscopy (SEM) is considered to be a technique faster than TEM, allowing the scanning of different samples at the same time, acquisition of pictures from patients or *in vitro* at the cellular and subcellular levels within 10 minutes, with fewer focus adjustments (Brahim Belhaouari et al., 2020; Colson et al., 2020). SEM allows imaging the entire infection cycle in Vero cells (Caldas et al., 2020) in 4.5 h (8 time points) instead of 8 h for TEM (Brahim Belhaouari et al., 2020). Cryo-electron microscopy is the best method to elucidate the viral ultrastructure (frozen samples, preservation of native conformation) but it is difficult to use it for patients’ samples as the virions are difficult to visualize. A better option to study the virus at larger scale is to use cell culture. Laue et al. infected Vero cells and observed ultrathin plastic sections, using TEM. The size of SARS-CoV-2 was similar to cryo-EM, slightly higher [100 nm, cryo-EM (90-97 nm, (Laue et al., 2021)], maybe because the shape was slightly different (oval instead of round). It is however possible that the differences observed were as a result of the cell culture conditions or difference in the viral strains.

Although EM is a powerful technique with very high resolution, being the only technique able to resolve assembled virions, it does have limitations.

### Challenges in EM

EM is actually not recommended for diagnostic, viral particles can be easily described incorrectly. Although different guides have been written on how to recognize SARS-CoV-2 particles in tissue (Neil et al., 2020; Akilesh et al., 2021; Hopfer et al., 2021), the task is not easy and requires a lot of expertise.

Moreover, the EM preparation process (preservation of autopsy samples, fixation) (Varga et al., 2020) can alter the viral structures and some subtle changes can be missed or the cellular organelles may be confused for virions (Neil et al., 2020).

Variable sizes of the virion were recorded in the same patient tissue, with the range depending on the study: 60-120 nm (Wang et al., 2020c), 80-100 nm (Mondeja et al., 2021), 90-140 nm (Werion et al., 2020). In general, the size of SARS-CoV-2 has been observed to be between 60 and 140 nm (**Table 1**). The different sizes observed could be due to the unsynchronized viral replication, and different stages of the viral cycle. Furthermore, the virus tends to cluster in cells leading to incorrect measurement.

**Table 1:** Light microscopy used for localization of SARS-CoV-2 proteins/RNA and Electron Microscopy for the detection of virion particles in different organs of SARS-CoV-2 infected individuals.

Organs	Light microscopy (LM): localization of viral proteins/RNA	Electron Microscopy (EM): localization and size of viral particles
Lung	(Satturwar et al., 2021): Spike protein mRNA: cytoplasmic (sense and antisense strands, lung tissue).	(Pesaresi et al., 2020): in vesicles or associated to internal membrane (Nardacci et al., 2021): In single-layered cytoplasmic compartments of different sizes (with multiple virions) or single virions in spherules (Wang et al., 2020d): cytoplasm of hepatocytes. 60-120nm
Upper respiratory tract	(Mondeja et al., 2021): (nasal epithelial cells) virus in the cell membrane (viral protein detection with a hyper-immune serum of the COVID-19 –convalescent patient) (Meinhardt et al., 2021): (olfactory mucosa) S protein: cytoplasmic, granular, partly perinuclear.	(Mondeja et al., 2021): 80-100 nm, in cell-free space: 80 nm
Gastrointestinal	(Xiao et al., 2020): cytoplasmic localization of nucleocapsid protein in gastric, duodenal, and rectum glandular epithelial cell	
Kidney	(Diao et al., 2021): Colocalization of NP and S antigens with ACE2 protein in the kidney tubules (Su et al., 2020): granular staining of NP in the nucleus or cytoplasm in tubular epithelium.	(Werion et al., 2020): in vacuoles or cisternae of the ER in PT cells. 90-140 nm with small dense irregular dots of 10–20 nm. Some particles budding into the ER lumen. (Su et al., 2020): cytoplasm, 65-136 nm, spikes: 20-25 nm (Farkash et al., 2020): individual viruses 65 - 91 nm. Vacuoles with double-membrane vesicles (partially assembled viruses)?. Mature appearing viruses in the cytoplasm in small arrays
Liver		(Wang et al., 2020d): 60–120 nm, cytoplasmic
CNS	(Paniz-Mondolfi et al., 2020): S protein in the cytoplasm of endothelial cells	(Paniz-Mondolfi et al., 2020): individually and in small cytoplasmic vesicles in endothelial cells, and bubbling from endothelial cells. 80-100 nm
Placenta	(Schoenmakers et al., 2021): S protein and SARS-CoV-2 RNA in the cytoplasm (syncytiotrophoblast cells of the placenta).	
Spleen and lymph nodes	(Feng et al., 2020b): NP only in cytoplasm	
Skeletal muscles		(Hooper et al., 2021): -degenerated cells: cytoplasmic clusters of viruses -viruses close to vesicles and membranes of ER and Golgi-like structures

NP, nucleoprotein; S, spike protein.

Although theoretically, SARS-CoV-2 should have the same morphology in cell culture and in patients’ tissues, this is not always the case for several reasons. As technicians in routine laboratories and virologists are unfamiliar with EM, mistakes can occur. Observations should be interpreted with caution because several cellular structures such as coated vesicles, secretory pathway, the endosomal network, multivesicular bodies (MVBs) and cross sections of the endoplasmic reticulum (ER), can mimic SARS-CoV-2 morphology leading to incorrect conclusions (Hopfer et al., 2021). An interesting observation is that the coated vesicles, MVBs and rough ER do not possess any electron dense dots characteristic of the nucleocapsids. Moreover, identification of SARS-CoV-2 in the cells cannot always be based on the presence of the “crown” because assembled virions occur within vacuoles and not directly inside the cytoplasm. Then, the corona spike will never be observed directly in contact with cytoplasm. Unless negative staining is used, the S protein forming the “corona” is not usually apparent.

**Figure 4:**
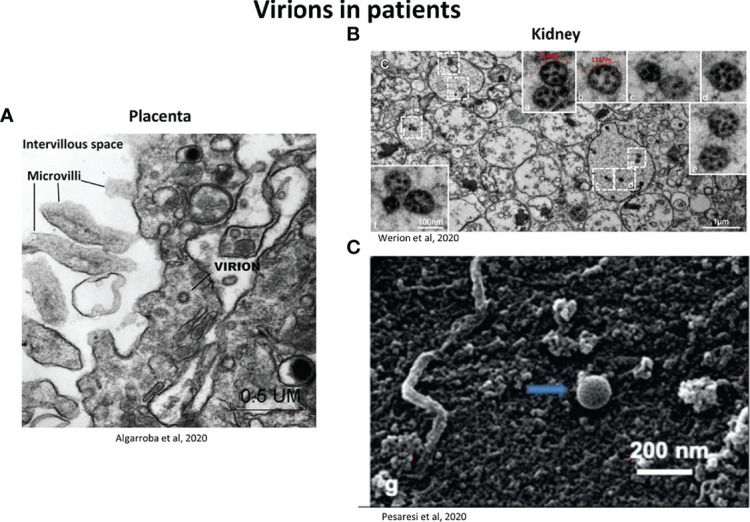
Representative electron micrographs showing the localization of SARS-CoV-2 virions in patients **(A)** Virion invading a syncytiotrophoblast (placenta) observed by TEM (50 000X). Scale bar: 0.5 µm (Algarroba et al., 2020). **(B)** Viral particles inside vacuoles inside the smooth endoplasmic reticulum (kidney section). Low magnification for the dashed-line boxes (scale bar: 1 µm) and high magnification for the solid line boxes (scale bar: 100nm) corresponding to the dashed-line boxes (a-f). Particles have irregular dense black dots inside and some (e.g. b and e) have crisp small rings (Werion et al., 2020). **(C)** SARS-CoV-2 located on the surface of a membrane of renal tissue, observed by SEM. Scale bar: 200 nm (Pesaresi et al., 2020).

**Figure 5:**
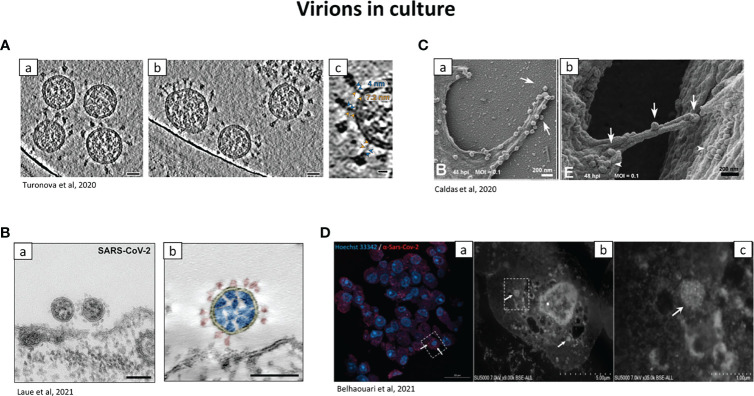
Representative electron micrographs showing the localization of SARS-CoV-2 virions in tissue culture. **(A)** Cryo–electron tomography of SARS-CoV-2 virions. a.b SARS-CoV-2 virions isolated from culture and centrifugated, observed by cryo-electron tomography. Scale bar: 30nm. c: zoom on the hinge of SARS-CoV-2, with the spike proteins. Blue and orange arrowheads: upper and lower legs, with their length indicated. Scale bar: 10 nm. (Turonova et al., 2020). **(B)** SARS-CoV-2 observed with Transmission EM. **(a)** TEM of Vero cells infected with SARS-CoV-2 (ultra-thin sections). Picture of viruses attaching to the surface of the cell. Scale bar: 100nm. **(b)** Transmission EM of a SARS-CoV-2 virus particle of at the surface of a Vero cell in an ultrathin plastic section (10 summed up digital slices of an electron tomogram). The colours were added manually: yellow=virus enveloping membrane, red=surface projection (spike, peplomer; trimeric S protein), blue=ribonucleoprotein (N protein and RNA). Scale bar=100 nm (Laue et al., 2021). **(C)** Virions at the surface of Vero cells by SEM. **(a)** Adherence of SARS-CoV-2 on phyllopodium-like extensions. **(b)** arrows indicate viral particles on a bridge between 2 infected cells. Arrowheads: aggregates of SARS-CoV-2 particles can be observed at the surface of both cells (Caldas et al., 2020). **(D)** Correlative light and electron microscopy of SARS-CoV-2 infected Vero E6 cells. a: confocal microscopy of a 100 nm-thick ultra-thin section (Z maximal projection). Blue: Hoechst 33342, nuclei; red: SARS-CoV-2 spike proteins. b,c: SEM of the ultra-thin section shown in **(A)** Higher magnification of the box shown in **(a)** Boxed region in b is shown in **(c)** arrows: clusters of SARS-CoV-2-like particles. Scale bars: a: 20 µm b: 5 µm c: 1 µm (Brahim Belhaouari et al., 2021).

Thus the term “virus-like particles” instead of virions should be used until definitive proof is obtained. A clue to differentiate virions from cellular components is the presence of electron dense dots inside the virion produced by the helical viral nucleocapsid (Neil et al., 2020). The use of good negative controls, such as tissues from non-infected individuals is important. **Figure 4** provides examples of virions visualized in patient tissue samples, for placenta (**Figure 4A**) and kidney (**Figures 4B, C**). Infection of cells in *in vitro* models is also a good alternative, to have a reference on the localization and morphology of the virions and to understand the viral replication cycle (Brahim Belhaouari et al., 2020; Laue et al., 2021) (**Figure 5**). SARS-CoV-2 particles were observed with electron tomography (**Figure 5A**), TEM, (**Figure 5B**) and SEM (**Figure 5C**).

As TEM on its own in not sufficient to determine easily the presence of the virus and IHC alone is not enough due to its low resolution and sensitivity, different options have been implemented.

### Options to Indisputably Detect the Virus

The solution is then to combine electron microscopy to antigen labelling, e.g. a paraffin embedded tissue section labelled for SARS-CoV-2 by IHC or *in situ* Hybridization (ISH) (Bullock et al., 2021). It is highly recommended to target different elements with different methods: the proteins (nucleocapsid or spike, by IHC or IF), the vRNA (by ISH, antisense probe for detection of viral replication or a sense probe for detection of viral messenger RNA; or Real-Time PCR (RT-PCR)) or directly the viral particles (morphology by EM) (Roberts et al., 2021; Von Stillfried and Boor, 2021).

ISH has a more complex methodology compared to RT-PCR so it would not be recommended for diagnosis (Lam et al., 2021; Von Stillfried and Boor, 2021). Lam et al., 2021, propose immunofluorescence as a first step of diagnostic (with a routine and large-scale/automated machine), followed by more sensitive RT-PCR, for the samples identified as negative by antigen detection method.

SARS-CoV-2 morphology was studied in different organs and cells (Laue et al., 2021) as well as the host organelles and their remodelling (Caldas et al., 2020) and an integrated view of the cell architecture reconstructed (Cortese et al., 2020).

Introducing electron microscopy together with *RNA-In Situ Hybridization* (RNA-ISH) allows the differentiation of cells that support viral replication from those that do not. Sense strand mRNA shows the presence of the virus, whereas detection of the anti-sense strand shows active viral replication, which occurs in the cytoplasm (Satturwar et al., 2021).

A novel method called Correlation Light-Electron Microscopy (CLEM) is a promising technique. It is an integrated technology allowing visualization of the same area of the slide using both electron microscopy and light microscopy, and then unequivocally confirming the presence of the virus (**Figure 5D**). Therefore it is important that multiple methods with high resolution are used to study the viral life cycle (Brahim Belhaouari et al., 2020).

Ultrastructural *in situ* hybridization of the virus was used by Cassol et al. (Cassol et al., 2020). Using metabolic labelling of new synthesized viral RNA and quantitative electron microscopy, Snijder et al. observed infected cells (Snijder et al., 2020). Two reliable techniques have been described to locate indisputably viruses in tissues: immunoelectron microscopy and a variant of correlative microscopy (Erman et al., 2021). Regarding the correlative microscopy, immunohistochemical labelling of SARS-CoV-2 capsid (+ diaminobenzidine chromogen DAB) was performed and combined with light and electron microscopy on paraffin section of nasopharyngeal. The same immunohistochemical protocol was applied on deparaffinized tissue fixed in osmium, embedded in Epon resin, and cut into semithin sections. Protocols for IHC and TEM were selected in order to keep a good accessibility of the antibodies to the epitopes and to have a highly specific reaction at an ultrastructural level. Presence of the virus was also confirmed by immunoelectron microscopy through immunogold labelling on ultrathin Lowicryl sections with primary antibodies against SARS-CoV-2 spike glycoprotein (Erman et al., 2021).

### Sensitivity and Specificity of the Techniques

A perfect concordance of the results is encountered between IHC and ISH, with qPCR confirming the result only when the viral load is high (El Jamal et al., 2021). However, it is difficult to determine if the virus still replicates or if the genome detected is from a past infection. The best way to confirm active replication of the virus would be to culture it but it is fastidious, requiring BSL-3 laboratory facilities, and does not always yield results (Gabbrielli et al., 2021).

Comparative studies showed that ISH has a sensitivity of 86.7% and a specificity of 100% compared to RT-PCR, IHC has a sensitivity of 85.7% and a specificity of 53.3% compared to RT-PCR (Massoth et al., 2021). A comparison between ICH and ISH showed 100% correlation between the 2 methods (Best Rocha et al., 2020; Remmelink et al., 2020).

### Discussion of the Microscopy Results

Negative results may imply degradation of viral DNA, RNA and proteins, or just a low viral load, viral clearance or lack of tropism for the organ. Moreover, positive results may be due to a real presence of the virus but also because of non-specific staining due to the tissue degradation. The general problem remains in the absence of a positive control, such as Vero cells infected with SARS-CoV-2, for comparison of the staining in microscopy (El Jamal et al., 2021). Also, positive result does not necessarily mean tropism for the organ, as the virus might be in transit to other organs.

The SARS-CoV-2 genome can be detected up to 1 month after death (Gabbrielli et al., 2021). The virus could not be detected by ISH but was encountered at very low level by RT-PCR in kidneys (Santoriello et al., 2020). In contrast, SARS-CoV-2 nucleocapsid can be detected by IHC in the respiratory tract several days after death (Skok et al., 2021). SARS-CoV-2 has been detected in lungs by IHC, 72 h to 96 h after death, in only 65% of patients, and there was a high heterogeneity of distribution of positive cells in the lung parenchyma (Remmelink et al., 2020).

Detection of the virus by IHC/IF is usually done in tissues with high viral load. The presence of the nucleocapsid (indirect immunofluorescence positivity) (but not RBD) correlates with the viral load (RT-PCR). On the other hand, no trend was seen for some samples, probably due to the quality of these samples (Lam et al., 2021).

Virus detection and distribution does not always correlate with histopathological findings. Biopsies of lungs with widespread injury may not always show the presence of virus (Deinhardt-Emmer et al., 2021). Histological detection is a detection tool which helps to retrace the temporal sequence of the events. SARS-CoV-2 infection appears to be transitory in a very early acute phase and triggers a cascade of inflammatory responses, which continues after clearance of the virus (El Jamal et al., 2021).

## Microscopy for Elucidation of the Viral Cycle and Interaction With the Host Cell

**Figure 6:**
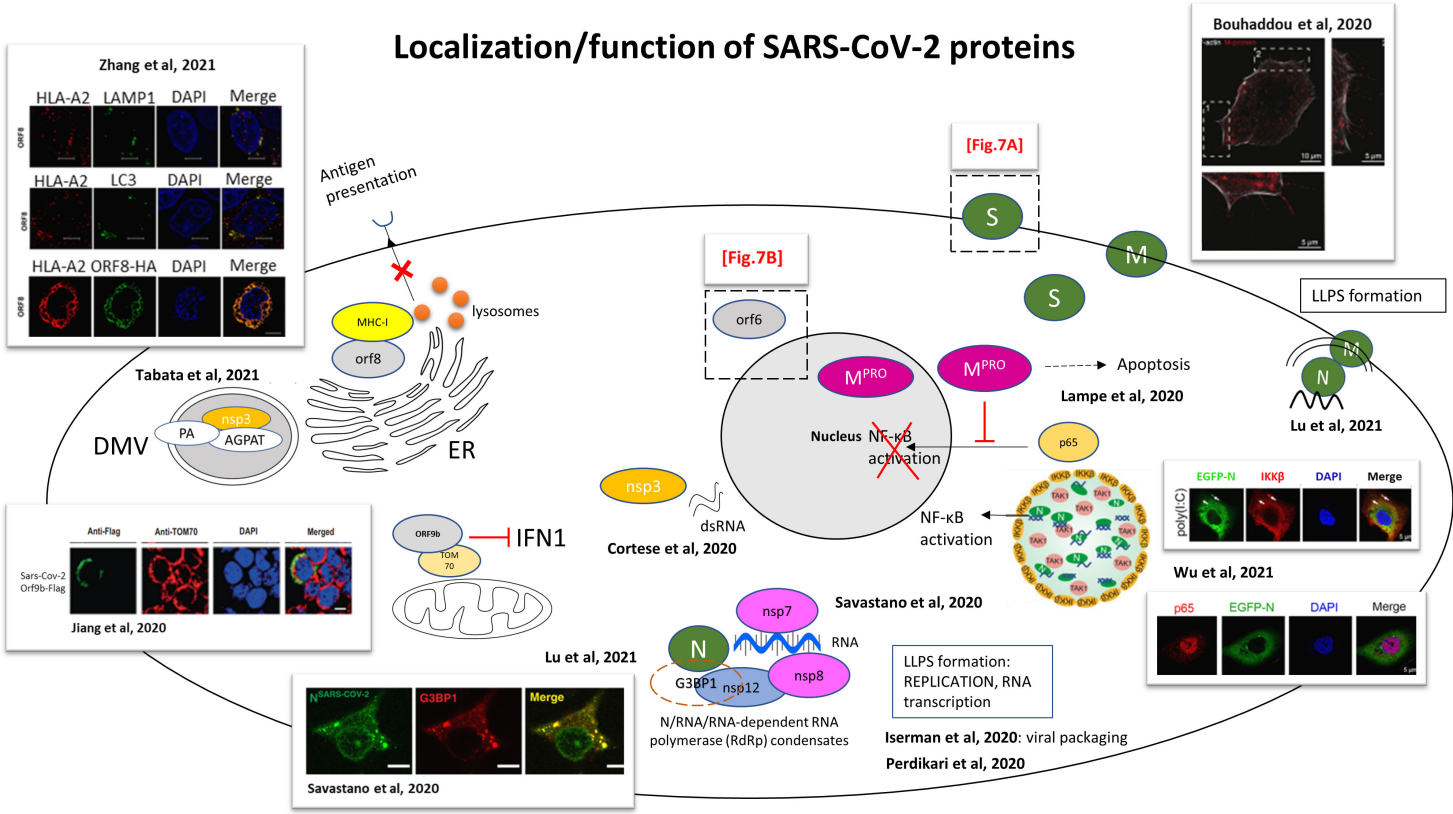
Functional studies for the localization and functions of SARS-CoV-2 proteins. Schematic representation of an infected cell and the localizations/functions of the different SARS-CoV-2 proteins. The drawings are based on the articles indicated in the figure. Dashed boxes: detailed regions in **Figures 7A**, **B** S: spike protein; M: membrane protein; N: nucleocapsid. M protein was encountered localizing with actin filaments (Bouhaddou et al., 2020). SARS-CoV-2 infected Caco-2 cells immunostained for F-actin (white) and M protein (red). M protein localizes along and to the tip of filopodia. 1 and 2, dashed box: magnification seen in pictures 1 and 2. Scale bar 10 µm; 5 µm in 1 and 2. SARS-CoV-2 main protease (Mpro) fused to HA tag (Mpro-HA) inhibits the nuclear translocation of the NF-κB subunit p65, blocking NF-κB activation (by abolition of NF-kB mediated gene transcription by IL-1β) (Wenzel et al., 2021). LLPS of SARS-CoV-2 N protein recruits TGF-beta-activated kinase 1 (TAK1) and IκB kinase **(IKK)** complex. Schematic representation from Wu et al. of SARS-CoV-2 N protein regulating NF-κB signaling pathway by recruiting TAK1 and IKKβ through liquid–liquid phase separation. Confocal picture: Huh7 cells stably expressing EGFP-N protein, treated with 1 μg/mL poly(I:C) for 6 h, followed by labelling IKKβ and DAPI. Scale bars, 5 μm (Wu et al., 2021b). Nucleocapsid protein LLPS concentrates SARS-CoV-2 replication machinery components: RNA-dependent RNA polymerase, non-structural protein nsp12, accessory sub-units nsp7 and nsp8 (required for transcription) concentrate with RNA/N droplets. Confocal picture: SARS-CoV-2 Nucleocapsid protein associates with stress granules, formed through LLPS to maximize replication efficiency. Colocalization of Alexa Fluor 488 labeled N-SARS-CoV-2 (green) with stress granule marker G3BP1 (red) in arsenite-treated digitonin-permeabilized HeLa cells. Scale bar: 10 µm (Savastano et al., 2020). N protein compacts viral RNA *via* protein-RNA LLPS, and interactions between N protein and host RNA-binding proteins are mediated by phase separation (Perdikari et al., 2020). SARS-CoV-2 N-protein undergoes liquid-liquid phase separation (LLPS) with the viral genome, and a model of viral packaging through LLPS is proposed (Iserman et al., 2020). SARS-CoV-2 double-stranded RNA, marker of viral replication, colocalizes with the viral protein nsp3 in the perinuclear region (Cortese et al., 2020). ORF9b colocalizes with mitochondrial TOM70 and blocks IFN1 production. Picture: Confocal microscopy of HEK 293T cells transfected by SARS-CoV-2 ORF9b-Flag. Staining with an anti-flag antibody (green) and an anti-TOM70 antibody (red). DAPI (blue): nuclei. Scale bar, 10 µm (Jiang et al., 2020). (Tabata et al., 2021): Confocal microscopy was used to show relocalization of AGPAT proteins to SARS-CoV-2 induced DMVs, colocalization of nsp3 (HA-nsp3) with PA (Phosphatidic acid) (GFP-PA) and AGPAT (acylglycerolphosphate acyltransferase) (GFP-AGPAT). CLEM was used to show formation of numerous DMVs. (Transient transfection of Huh7 cells with SARS-CoV-2 HA-nsp3-4-V5 expression construct and EGFP-PABD-Raf1-WT construct; or transfection with AGPAT2-GFP expressing plasmid and HA-nsp3-4-V5 expression construct). SARS-CoV-2 ORF8-HA co-localizes with MHC-I (HLA-A2), mediating MHC-I downregulation *via* degradation. Confocal picture: co-localization of HLA-A2 (red) with LAMP1 (green) lysosomes, with LC3 (green) (marker of autophagosomes) and with SARS-CoV-2 ORF8-HA (green). Scale bars, 5μm. HEK293T cells were transfected with ORF8-HA expressing plasmid (Zhang et al., 2021b). SARS-CoV-2 N protein forms condensates with viral RNA and membrane associated M protein for packaging of the viral RNA (Lu et al., 2021).

In designing therapeutic strategies, it is necessary to have a good understanding of the viral cycle, the trafficking of viral proteins and their interactions with the host. Functional studies in cell culture have elucidated different mechanisms induced by SARS-CoV-2 proteins (**Figure 6**). Although EM gives a high resolution, the treatment of samples can damage the virus. Moreover, sensitivity can be a problem, some tissue having a very low infection rate. Also, by the time of the sampling, the immune system can have already cleared the virus from the tissue, and the amount of material is limited. It is then important to use light microscopy techniques and to establish models to study the viral cycle. Confocal microscopy is a very popular technique, non-invasive, which requires less sample preparation than EM. Also, live cell imaging can be performed.

In order to track the viral proteins in the organelles, cells are stained with viral and host specific reagents. Cells are fixed and stained with available antibodies [anti-SARS-CoV-2 spike, or Nucleocapsid antibodies) (**Table 2, Supplementary**)] and/or with antibodies targeting various cellular organelles. Alternatively, live imaging can be done with viruses tagged with either green fluorescent protein (GFP) or mcherry for direct fluorescence (Yi et al., 2020). A common technique used is confocal microscope, giving images of focal planes to provide a precise localization of the viral elements within the cell, results enhanced by the use of higher resolution microscopy such as Stimulated Emission Depletion (STED). Using microscopy, analogies and differences were encountered between SARS-CoV and SARS-CoV-2 and these could be responsible for their difference in pathogenicity (Laue et al., 2021).

### Cell Entry

**Figure 7:**
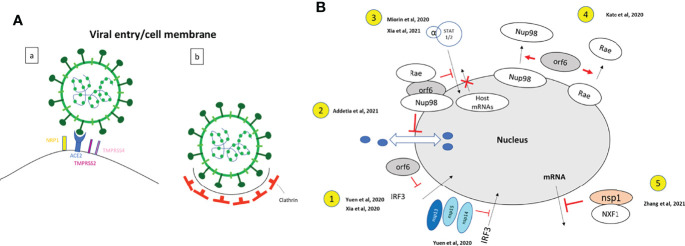
Functional studies for the localization and functions of SARS-CoV-2 proteins. Enlargement of the dashed boxes of **Figure 6**. **(A)** Viral entry: **(a)** Drawing based on data from (Cantuti-Castelvetri et al., 2020; Zang et al., 2020; Storti et al., 2021): Neuropilin-1 (NRP1) facilitates SARS-CoV-2 (Lentiviral particles pseudotyped with the SARS-CoV-2 S protein) cell entry and infectivity (Cantuti-Castelvetri et al., 2020). SARS-CoV-2 spike protein binds to ACE2 receptor, with colocalization of ACE2 and RBD of S protein (Storti et al., 2021); TMPRSS2 and TMPRSS4 facilitate SARS-CoV-2 spike fusogenic activity and promoted virus entry into host cells (Zang et al., 2020). (HEK293 cells were infected with VSV-SARS-CoV-2). **(b)**. Drawing based on data from (Storti et al., 2021). Clathrin has a role of mediator of late entry endocytosis of SARS-CoV-2 (infection of Vero E6 cells by SARS-CoV-2 isolated from patients). Strong colocalization of S protein with clathrin; confocal, Airyscan, dSTORM-TIRF and τ-STED techniques are used to visualize single virions overlapping with clathrin. **(B)** Nucleus: Enlargement of the dashed box **Figure 6**. Drawings based on literature indicated on the figure. 1. SARS-CoV-2 nsp13, nsp14, nsp15 and ORF6 (nsp13-FLAG, nsp14-FLAG, nsp15-FLAG and ORF6- FLAG) act as potent interferon antagonists and prevent IRF3 nuclear entry. A549 cells were transfected with ORF6-expressing plasmid (Xia et al., 2020; Yuen et al., 2020). 2. SARS-CoV-2 ORF6 interacts with Rae and Nup98 and disrupts bidirectional nuclear cytoplasmic transport. (GFP-ORF6 expressing plasmid transfected in 293T cells) (Addetia et al., 2021). 3. SARS-CoV-2 ORF6 interacts Nup98 to block STAT nuclear import and interfere with interferon signaling. (HEK293T transfected with ORF6-HA expressing plasmid) (Miorin et al., 2020; Xia et al., 2020). 4. SARS-CoV-2 ORF6 dislocates Nup98 and Rae from the nuclear pore complex and relocalizes them to the cytoplasm (Hek293T cells transfected with ORF6-GFP expressing plasmid) (Kato et al., 2021). 5. SARS-CoV-2 Nsp1 disrupts the mRNA export machinery, by interacting with the host mRNA export receptor heterodimer NFX1-NXT1. (Hek293T cells transfected with GFP-Nsp1 expressing plasmid) (Zhang et al., 2021a).

SARS-CoV-2 uses the human angiotensin converting enzyme 2 (hACE2) receptor to enter cells (**Figure 7A**). Multiple studies showed the colocalization of SARS-CoV-2 spike protein or TMPRSS2 with ACE2 receptor (Wang et al., 2020c; Bayati et al., 2021; Karthika et al., 2021; Liu et al., 2021; Muller et al., 2021). The SARS-CoV-2 C-terminal domain (CTD), which has high sequence identity with SARS-CoV receptor binding domain (RBD), co-localized with GFP-hACE2 receptor, this interaction between the proteins was demonstrated using crystallography (Wang et al., 2020c). In cooperation with hACE2, TMPRSS2 and TMPRSS4 (plasma membrane–associated type II transmembrane serine protease) mediate the fusion of SARS-CoV-2 with the cell membrane. An *in vitro* system together with confocal microscopy revealed that hACE2 and TMPRSS2 proteins induce SARS-CoV-2 S cell-cell fusion and the formation of syncytia in enterocytes, more intensely than when TMPRSS4 is expressed (Zang et al., 2020). This system consisted on a co-culture of 2 different types of cells: donor cells, which express SARS-CoV-2 S protein transfected with dtomato, and target cells, which express hACE2 with either TMPRSS2 or TMPRSS4 and transfected with GFP. TMPRSS4 can only act in *trans* whereas TMPRSS2 can act both in *cis* and in *trans*, leading to the formation of larger syncytia (Zang et al., 2020). The spike protein was involved in synapse-like intercellular contacts, leading to cell-cell fusion and formation of syncytia (Sanders et al., 2021). Additionally, neuropilin 1 (NRP1), which co-localizes with S protein in olfactory epithelium cells, was shown to be an additional factor to favour cell entry (Cantuti-Castelvetri et al., 2020).

After recognition of the receptor by the spike protein, endocytosis and formation of endosomes are very important for infection (Liu et al., 2020b). SARS-CoV-2 infection depends on endosomal pH and cholesterol lipid rafts (Li et al., 2021b; Lv et al., 2021; Zhang et al., 2021b). The viral membrane fuses with the endosome membrane and the virus releases its RNA in the cytosol.

### Viral Replication

Using a combination of confocal and super-resolution techniques, it was demonstrated that infection with SARS-CoV-2 induces a complete reorganization of the organelles of infected cells, including ER, peroxisomes, mitochondria and the secretory apparatus (Cortese et al., 2020). This cellular reorganization, which is a major consequence of the infection, promotes viral replication. Coronaviruses use host membranes to create replication organelles (RO) and double membrane vesicles (DMV) as replication sites (Wolff et al., 2020). Confocal imaging showed the ER reorganization with tubular ER protein Reticulon 3 (RTN3), double-stranded RNA (marker of viral replication) and nsp3 protein in the perinuclear region, with accumulation of peroxisomes close to the dsRNA, revealed by STED microscopy (Cortese et al., 2020).

Infection also changes the morphology and localization of mitochondria, appearing thinner, with swollen cristae and matrix condensation. The ATP synthase subunit 5B, essential for energy production, is downregulated, possibly inducing a decrease of cellular metabolism (Cortese et al., 2020; Perez-Leal et al., 2021). Mitochondrial dysfunction was observed, with membrane depolarization, permeability transition pore opening and increased ROS release (Shang et al., 2021). Mitophagy was initiated for mitochondrial quality control and virus clearance, but was inhibited by SARS-CoV-2 (Shang et al., 2021), which constitutes a viral anti-autophagy strategy. Mitochondrial damages (Soria-Castro et al., 2021), abnormalities (Dodig et al., 2022) and fragmentation (Clough et al., 2021) were observed in tissue samples, as well inhibition of mitochondrial function (De La Cruz-Enriquez et al., 2021).

Focused Ion Beam-Scanning Electron Microscope (FIB-SEM), together with automated segmentation and machine learning for organelle recognition can reveal the 3D structure of entire infected cells (Cortese et al., 2020). Together with electron tomography, an interconnected DMV network can be observed following SARS-CoV-2 infection, with DMVs connected to the ER through ER connectors (Cortese et al., 2020). The virus actually hijacks the autophagy machinery (Samimi et al., 2021) in order to create these DMVs, which are used as replication organelles (Twu et al., 2021). The Golgi apparatus, which appears to be fragmented, is the viral assembly site and is involved in budding events (Cortese et al., 2020). The tight proximity of DMVs with secretory pathway (ER, Golgi) allows a perfect coordination between RNA synthesis, translation and viral packaging (Cortese et al., 2020). Host cell lipids also contribute to SARS-CoV-2 and DMVs formation. Using confocal microscopy and CLEM, phosphatidic acid (PA), produced by acylglycerolphosphate acyltransferase (AGPAT), and AGPAT have been colocalized with nsp3 in nsp3-4-induced DMVs (Tabata et al., 2021) (**Figure 6**).

### Viral Packaging and Egress

Usually, autophagy plays a role in the elimination of the viruses by fusion of the autophagosomes with the lysosomes. In the case of SARS-CoV-2, viral nsp6 (Sun et al., 2022) and ORF3a block autophagy by blocking autophagosome-lysosome fusion (Miao et al., 2021; Perez-Leal et al., 2021; Zhang et al., 2021c) and then promote viral egress through lysosomal exocytosis (Chen et al., 2021; Scherer et al., 2022). The virus uses small vesicles which fuse with the plasma membrane and release the viruses into the extracellular space (Eymieux et al., 2021).

By the means of confocal, STED microscopy and Fluorescence Recovery After Photobleaching (FRAP) experiments for physical characterization of the proteins, it has been proposed that N protein participates in viral packaging through Liquid-liquid phase separation (LLPS) (Iserman et al., 2020), stimulated by viral gRNA (Perdikari et al., 2020) and associates to stress granules (Savastano et al., 2020). These RNA/protein condensates recruit the SARS-CoV-2 RNA-dependent RNA polymerase complex (formed by the SARS-CoV-2 nsp12, nsp7 and nsp8 (Savastano et al., 2020) (**Figure 6**). This complex leads to an efficient transcription of viral gRNA. Moreover, the use of 2 types of probes for RNA-ISH allows the differentiation between the presence of the virus in the cell (sense probe) and active viral replication (antisense probe) (Satturwar et al., 2021). Thus, the replication site was located in the cytoplasm of infected cells (Satturwar et al., 2021). Another model has been suggested for the packaging of the nucleocapsids, requiring the 3 elements N, M and gRNA, where the N protein separates with each of the M protein and gRNA (Lu et al., 2021) (**Figure 6**). Alternatively, virions can be encountered in MVBs, which could be an alternative route of secretion (Satturwar et al., 2021).

### Cytoskeleton Reorganization

Cytoskeleton reorganization is also an important characteristic of infected cells. Actin accumulates at the plasma membrane. Infection induces an increase in the number and the length of filopodial extensions, where M and N proteins accumulate. SEM and TEM confirmed the presence of assembled viral particles budding from these filopodia, especially at the tips (Bouhaddou et al., 2020) (**Figure 6**). Large intracellular vesicles containing spike protein surrounded by an actin ring were captured by STED microscopy (Caldas et al., 2020). Intermediate filaments also play a role, surrounding perinuclear inclusions containing dsRNA. Super-resolution STED microscopy captured rearrangement of the cytoskeleton after infection of the cell with high precision. Spike protein was encountered in large intracellular vesicles surrounded by an actin ring. DsRNA was located in perinuclear inclusions surrounded by intermediate filaments forming a cage, this cage was surrounded by microtubules (Cortese et al., 2020).

### Interference With Immunity

During an infection, viral pathogen-associated molecular patterns (PAMPs) activate transcription factors, which are translocated to the nucleus, leading to the production of interferon (IFN), which is important in the antiviral response. However, SARS-CoV-2 contains various proteins, which prevent IFN production. This viral protein interference with IFN pathways was observed by confocal microscopy, mainly by co-localization studies, with viral proteins fused with haemagglutinin (HA), FLAG tag (DYKDDDDK) or GFP-tag. Such experiments aim to understand this interference in order to find therapeutics that restore the IFN response in COVID-19 patients.

SARS-CoV-2 ORF6, like SARS-CoV, is a strong IFN antagonist. SARS-CoV-2 nsp13 (helicase), nsp14 (exonuclease), nsp15 (endoribonuclease) and ORF6 (accessory protein) suppress Interferon Regulatory Factor 3 (IRF3) nuclear translocation and then IFN production (Yuen et al., 2020) (**Figures 6** and **7B, 1**). ORF6 co-localizes and interacts with Nup98-Rae1 at the nuclear pore complex also prevents the nuclear import of STAT1/STAT2 and then the transcription of IFN-stimulated genes (ISGs) (Miorin et al., 2020). However, it is not the case of SARS-CoV-2 Papain-like protease (PLpro), which lost its anti-IFN effect compared to SARS-CoV PLpro (Yuen et al., 2020). RIG1-like receptors, which are intracellular pattern recognition receptors, detect viral RNA, activate adapter proteins MAVS/TRAF3/TRAF6/TOM70 on mitochondria, which activate IRF3 and induce IFN response. SARS-CoV-2 ORF9b (alternative ORF with the nucleocapsid), like SARS-CoV ORF9b, colocalizes with the adapter protein TOM70 on the mitochondrial membrane, and inhibits IFN-I production **Figure 6** (Jiang et al., 2020). Accessory protein ORF8 can downregulate MHC-I (Zhang et al., 2021b). When ORF8 was expressed in cells, expression of MHC-1 at the cell surface was impaired and redistributed in the cytoplasm, where it co-localized with ORF8 and the lysosomal marker LAMP1. Also, ORF8 co-localized with ER marker calnexin, and a fraction co-localized with autophagosomes. These observations lead to the hypothesis that ORF8 interacts with MHC-I in the ER, brings it to autophagosomes and lysosomes for degradation. As a consequence, MHC-I is expressed at lower levels on the cell surface and the infected cell cannot be eliminated by CTL in COVID-19 patients, hence the development of chronic disease (Zhang et al., 2021b). Down-regulation of MHC-I is not observed with SARS-CoV ORF8, probably because of the low homology of 26% between ORF8 of the two viruses (Iatsenko et al., 2021).

N protein binds to viral RNA and undergoes LLPS, leading to NF-κB activation (Wu et al., 2021b) (**Figure 6**). The Mpro protein, SARS-CoV-2 main protease, blocks the nuclear entry of the NF-κB subunit p65, preventing the NF-κB activation (Wenzel et al., 2021) (**Figure 6**).

ORF6 has been involved in different processes regulating the immune response (**Figure 7B**). SARS-CoV-2 ORF6, like SARS-CoV, is a strong IFN antagonist. Besides its role in blocking IRF3 nuclear import (Xia et al., 2020; Yuen et al., 2020), ORF6 associates to the nucleoporin nup98 and nuclear export factor Rae, leading to an accumulation of host mRNA in the nucleus and blocking the nuclear import and export of host proteins (Addetia et al., 2021) (**Figure 7B, 2**). This interaction of ORF6 with Nup98-Rae1 at the nuclear pore complex prevents also the nuclear import of STAT1/STAT2 (Miorin et al., 2020; Xia et al., 2020) and then the transcription of IFN-stimulated genes (ISGs) (Miorin et al., 2020) (**Figure 7B, 3**). Kato et al. demonstrated that ORF6 delocalizes the localization of Nup98 and Rae from the nuclear ring to the cytoplasm (Kato et al., 2021) (**Figure 7B, 4**). The non-structural protein 1 (nsp1) was localized in the nucleus and cytoplasm of infected cells and can also alter the localization of the nuclear pore complexes proteins. Indeed, nup93 interacts with nsp1 and is shifted to the nucleoplasm (Gomez et al., 2019). Nsp1 was also shown to interact with the host messenger RNA (mRNA) export receptor NXF1 -NXT1, which prevents nuclear export of cellular mRNAs (Zhang et al., 2021a) (**Figure 7B, 5**).

### Mechanisms Molecularly Associated With Aging and Driven by Viral Infection

Among age-related diseases, cancer [molecular similarities with high level of cytokine, type I interferon, androgen receptor, and immune checkpoint signalling (Zong et al., 2021)], diabetes, cardiovascular disorders, and neurodegenerative diseases can be driven by SARS-CoV-2 infection.

The high level of immune response and the low level of antiviral response (Neufeldt et al., 2022) does not contribute to a favourable prognosis and leads to injuries of multiple organs, including brain, lungs and heart. SARS-CoV-2 infects cardiomyocytes and induces infiltration of immune cells. The cytokine storm generated is then responsible of myocarditis and cardiac dysfunction (Bailey et al., 2021; Gauchotte et al., 2021).

Invasion of SARS-CoV-2 in the brain of COVID-19 patients might implicate neurological complications. Phosphorylated α-synuclein (α-syn), which accumulates in the brain of Parkinson disease patients, is also encountered in biopsies of post-mortem SARS-CoV-2 infected patients (Szabo et al., 2022).

Among hallmarks of aging observed by microscopy techniques, mitochondrial dysfunction is an important one. Epigenetic alterations might be involved. SARS-CoV-2 has been shown to induce epigenetic-mediated metabolic reprogramming in a murine mouse model with the human ACE2 transgene (Li et al., 2021a). Finally, senescence could be increased after infection of AT2 cells with SARS-CoV-2 (Evangelou et al., 2022).

## Additionnal High Resolution Microscopy Methods Used for Studying SARS-CoV-2

The urgency for answers in the SARS-CoV-2 research field has stimulated the development of new preparation, microscopy and detection methods. The standard immunohistochemistry methods, with paraffin embedded thin sections, require a long time of preparation and imaging. Furthermore, these techniques can damage the samples because of multiple physical sections, are very costly and give only a 2D representation of the tissue. Nowadays, new high-end techniques, called “virtual histology” allow a 3D representation of a high volume of infected tissue (>200mm^3^ (Zaeck et al., 2021), the standard techniques indicating only the presence/absence of the virus), with only optical sectioning, and this prevents tissue damage. 3D imaging is made possible by the combination of tissue optical clearing (TOC) techniques, which make the tissue transparent, and specific microscopes such as the Light-Sheet Fluorescence Microscope (LSFM) (Matryba et al., 2019). In the following two studies, lung tissue from patients who died from COVID-19 was analysed. Propagation-based X-ray phase contrast tomography (PC-CT) has been used to reconstruct the architecture of unstained lungs (Eckermann et al., 2020), alveolar damage and lymphocyte infiltration. Morphological changes have been imaged in 3D, such as diffuse alveolar damage, with hyaline membranes and inflammation. This equipment gives an isotropic resolution but needs very good optimization before acquisition of pictures.

LSFM (Glaser et al., 2017) has emerged and constitutes an improvement of confocal microscope. It has been used in cell and developmental biology where the specimen is embedded in agarose, illuminated with a thin light sheet and the emitted photons are collected perpendicularly. The sample rotates and images are captured from different views, then 3D images can be reconstituted.

Compared to confocal microscopy, in LSFM, only the focal plane is illuminated, which reduces photodamage and stress in living samples. Moreover, the samples are scanned with a plane of light (instead of a point in confocal), so the speed of acquisition is increased (10-1000 times). Indeed, the signal-to-noise ratio is greatly improved. This technique can be very cost effective as one can build one’s own microscope in the laboratory. An improvement of the LSFM lead to the inverted selective plane illumination microscopy (iSPIM), where an inverted microscope includes 2 perpendicular water objectives so the LSFM is then compatible with coverslips (Kumar et al., 2014). Finally, iSPIM was modified, leading to the dual-view inverted selective plane illumination microscopy (diSPIM) (Wu et al., 2013; Kumar et al., 2014), where a second view of illumination of the sample has been added. DiSPIM then allows high speed of acquisition (200 images/s), reduces photobleaching, and increases spatiotemporal resolution compared to spinning disk confocal microscope and Bessel beams methods. However, diSPIM has a major disadvantage and limitation, the huge dataset generated.

**Figure 8:**
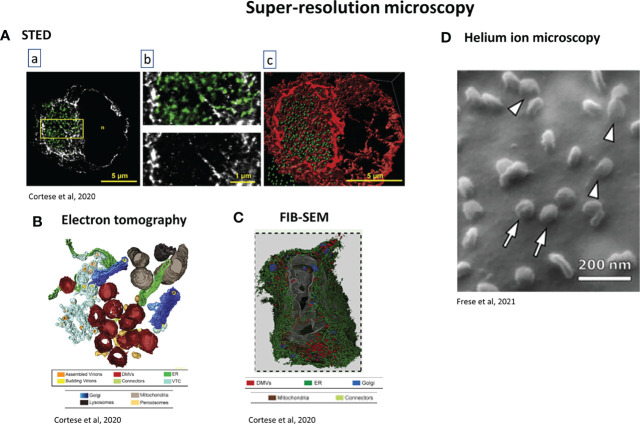
Examples of super-resolution techniques used for studying SARS-CoV-2. **(A)** STED microscopy. A549-ACE2 cells were infected with SARS-CoV-2 for 16 h, fixed and stained. **(a)** Green: dsRNA, gray: vimentin. Scale bar: 5µm. The region in the yellow box is magnified in b, Scale bar: 1µm. **(c)** 3D rendering of the dsRNA and vimentin signals after z-stacking. Scale bar: 5µm (Cortese et al., 2020). **(B)** 3D rendering of electron tomography of Calu-3 infected cells. Colour code at the bottom of the picture. It shows the spatial coupling of SARS-CoV-2 replication and assembly sites mediated by close proximity of DMVs, vesicular-tubular compartment and Golgi apparatus (Cortese et al., 2020). **(C)** FIB-SEM analysis of whole-cell volume of a SARS-CoV-2-infected Calu-3 Cell. Cells were infected 24h before fixation. 3 dimensional rendering of one infected cell. Colour code at the bottom of the figure. This reveals the network of DMVs and ER (Cortese et al., 2020). **(D)** Helium ion microscopy of SARS-CoV-2 infected cells. Arrowheads: viruses which appear to be bound to the cell membrane; arrows: viruses which appear to lie on the top of the cell membrane (Frese et al., 2021).

Li et al. (Li et al., 2020) used a diSPIM technique adapted from Wu et al. (Wu et al., 2013) to show, with submicron resolution, pulmonary damage, microangiopathy and the presence of megakaryocytes following SARS-CoV-2 infection. Light-sheet microscope and fluorescent H&E analogue staining were combined (TO-PRO-3 for nuclear contrast and Eosin-Y for cytoplasmic/stromal contrast) to obtain a “pseudo-colour” image. However, the analyses conducted by Li et al. (Li et al., 2020) and Eckerman et al. (Eckermann et al., 2020) were unable to provide a complete representation of the infection. There is no direct visualization of the viral particles and infection loci, which would give a better understanding of the host-pathogen interactions. This, was achieved by correlated LSFM-CLSM (Zaeck et al., 2021) (LSFM for large scale imaging and CLSM for subcellular details) with specific anti-SARS-CoV-2 antibodies, allowing quantifiable results. The viral infection foci were visualized in upper respiratory tract in ferrets with anti-nucleocapsid antibody. An integrative imaging approach was realized using a combination of light microscopy (confocal and super-resolution STED microscopy) and electron microscopy (TEM, electron tomography) (Cortese et al., 2020) (**Figures 8A, B**). These methods demonstrated the effect of SARS-CoV-2 infection on the host and the re-organization of different cellular organelles such as peroxisomes, mitochondria, the ER and cytoskeleton network. Viral dsRNA and replication sites were localized with a fluorescent probe by STED with a resolution of 70-90 nm. Higher details of cellular structures and detection of viral particles were given by TEM, 3D imaging techniques such as electron tomography and FIB-SEM (**Figures 8B, C**).

FIB-SEM, which belongs to the group of techniques called “volume electron microscopy”, gives 3D reconstruction of the cell architecture with a resolution of 5-20 nm from micron-thick specimens, with the advantage of not disrupting the tissue (Risco et al., 2014) and in comparison to TEM or electron tomography, larger volumes of sample can be imaged. A protocol has been written to help virologists/cell biologists using FIB-SEM. Using SARS-CoV-2, it showed the interaction of the virus with the cell membrane, and allowed the quantitation of viral densities and cell curvature (Baena et al., 2021).

Another combination of microscopy techniques was used to study the entry and egress of SARS-CoV-2 and its variants (Storti et al., 2021). At the cell level, confocal and Total Internal Reflection Fluorescence (TIRF) were used. TIRF is an appropriate technique to study events at the cell membrane, showing especially clathrin mediated endocytosis. Superresolution techniques Airyscan, STORM and Single Molecule Localization Microscopy (SMLM) were used to track single viruses, their size and their interactions with the cells.

Helium ion microscopy (HIM) can be used as a complementary technique for virus-cell interaction, with a resolution of up to 1.3 nm. It does not require any coating and allowed the differentiation between a SARS-CoV-2 virion binding to the cell membrane and a one just lying on the top of the membrane (Frese et al., 2021) (**Figure 8D**).

## The Need of Developing Rapid SARS-CoV-2 Screening Methods Using Microscopy

High-scale, rapid, sensitive and cost-effective screening tests are essential to limit the spread of a virus. Nowadays, the real-time polymerase chain reaction (RT-PCR) is the standard method for detection of infection. However, several issues have arisen, including false-negative and false-positive results. Microscopy can be used as a tool for the development of rapid detection assays as numerous techniques are available in this field. Lam et al., 2021 demonstrated the potential of immunofluorescence as a simple diagnostic tool; however, due to sensitivity problems, other detection methods have to be developed (Arevalo-Rodriguez et al., 2020).

An assay for naked-eye detection of SARS-CoV-2 (Moitra et al., 2020) has been described, where gold nanoparticles were capped with thiol modified antisense oligonucleotides specific for SARS-Cov-2 N gene. In the presence of their target SARS-CoV-2 RNA sequences, the gold particles agglomerate and a change in their hyperspectral signature apperance, which is observed with hyperspectral-enhanced dark field microscopy and electron microscopy.

A single molecule assay, which does not require any enzyme, usually used in conventional methods such as RT-PCR, was developed for a direct detection of SARS-CoV-2 RNA from patient’s samples (Furth et al., 2021). It consists a first step of *in vitro* hybridization of the viral RNA with 2 types of DNA probes: biotin-labelled capture probes and fluorophore-labelled detection probe. The complexes are then immobilized on a steptavidin-coated coverslip and viewed by TIRF microscopy. Each spot corresponds to 1 viral RNA and then allows quantitative analyses.

Already used with EM images (Li et al., 2021b; Rodriguez et al., 2021), machine learning is useful to directly detect unlabelled viruses for routine diagnosis (Goswami et al., 2021). Imaging of the virus was achieved using an ultrasensitive interferometric method and artificial intelligence. Images were taken with a spatial light-interference microscopy (SLIM) module added to a traditional phase-contrast microscope, and epifluorescence microscope. Images were used to train a U-Net convolutional neural network and a semantic segmentation map was generated. Shiaelis et al., 2020, proposed a method which combines single particle tracking and deep learning, with a diagnosis in less than 5 minutes (Shiaelis et al., 2020).

An alternative method to the detection of the antigens or the vRNA, is the detection of the specific anti-SARS-CoV-2 antibodies in human sera (Pape et al., 2021), semi-quantitative analysis (Williams et al., 2021).

## Model Systems for Study of SARS-CoV-2: Spheroids, Organoids, Microfluids Associated to Microscopy

*In vitro* models are essential in the life sciences (Kim et al., 2020), especially in virology (Sridhar et al., 2020). The advantages and disadvantages of each model for studying SARS-CoV-2 have been described and discussed (Rosa et al., 2021). Cell-based assays are cost-effective and allow the control of experimental variables, and have been used for drug-testing. However, they are not representative of physiological conditions. Tissue samples and spheroid/organoids recapitulate *in vivo* conditions well. Scaffold techniques, or scaffold-free techniques like bioprinting, microfluidics, have been developed in order to recreate 3D environment (Shpichka et al., 2020; De Melo et al., 2021), for the study of pathogens but have not been tested for SARS-CoV-2 yet. As laboratories try to avoid animal testing, and 2D models do not give a good representation of the infectivity, spreading and drug efficacy, these 3D models are a good alternative. All these techniques are powerful tools used in combination with microscopy, such as LSM and brightfield microscopy.

Organoids can derived from human induced pluripotent (iPSCs) or multipotent stem cells (Adipose-derived mesenchymal stem cells, ASCs), from embryo, foetal or adult origin. It is an alternative to animal models, which are costly, difficult to manipulate and not always adapted to human pathologies. Human organoids are thus more convenient for testing and analysing viral infections. They confer three-dimensional (3D) structure with the complexity of an organ to mimic *in vivo* conditions, which a monolayer of cells does not allow. A pluripotent stem cell-based platform has been created, giving rise to different organs from human iPSCs and used to study SARS-CoV-2 tropism (Yang et al., 2020). Organoids are commonly visualized with confocal microscopy. Although the penetration depth of fluorescence is quite limited considering that the organoid can reach 100 µm thickness depending on the type, it is enough to acquire good pictures.

**Figure 9:**
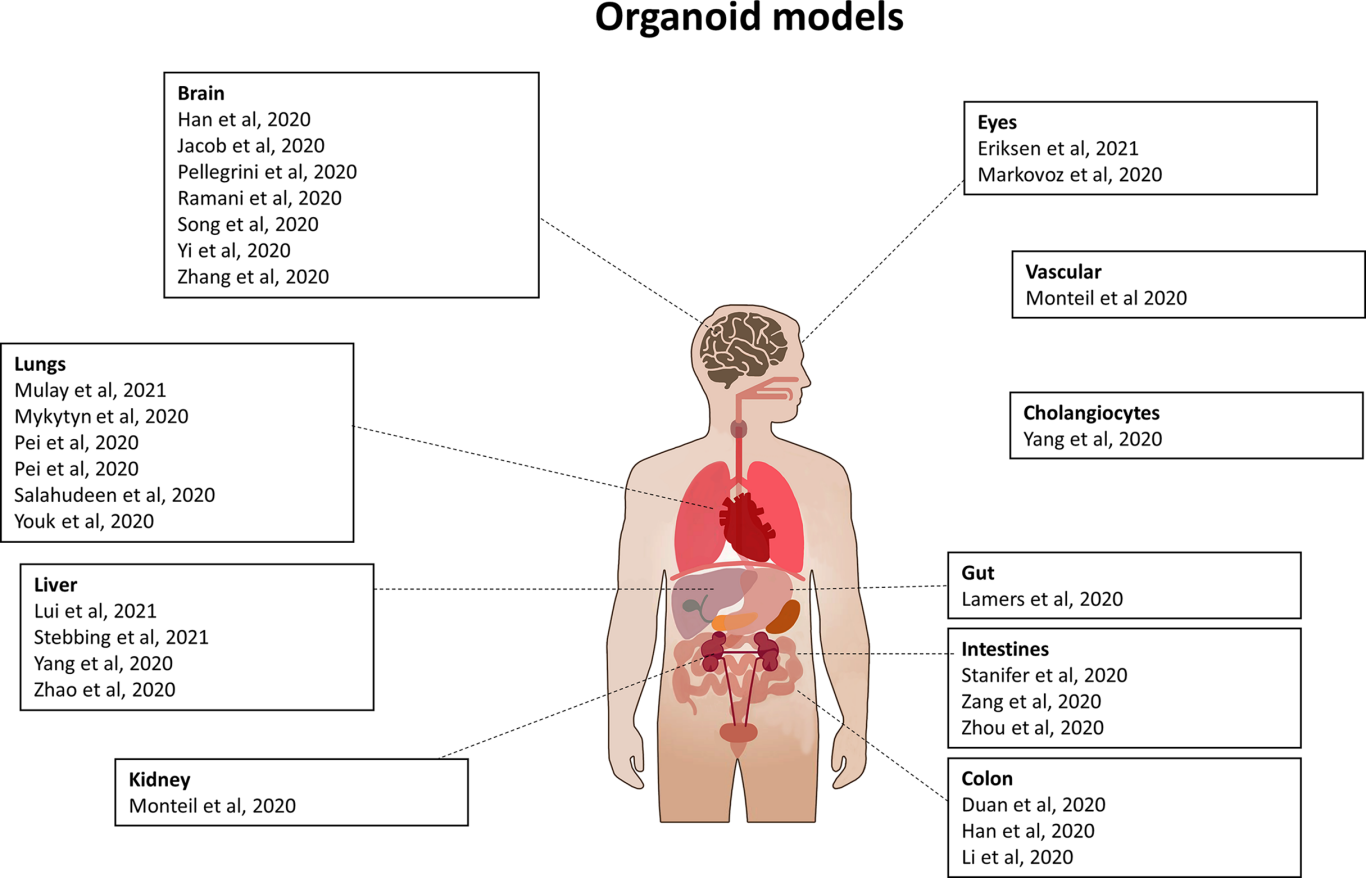
Organoid models used for SARS-CoV-2 study. List of organoids which have been used to study SARS-CoV-2 infection, viral life cycle and/or for drug testing.

SARS-CoV-2 infects multiple organs. Scientists are trying to recreate each infected organ *in vitro*, so as to have convenient model systems, in order to study the virus, its mechanisms, its pathogenicity and the localization of its proteins (**Figure 9**). The first step is to validate these organoids and ensure that they can be used as a model of infection by SARS-CoV-2.

Different types of cells can be tested to determine the ones permissive to infection, to compare their infection rate [eg duodenum *vs* ileum *vs* colon (Zang et al., 2020)], or to determine the exact location of the virus (Yi et al., 2020). For this purpose, pseudotyped SARS-CoV-2-mcherry viruses were used to infect brain organoids for example. The viruses could be detected in the axon of neurons. Zhang et al. (Zhang et al., 2020) created a platform derived from iPSCs (Mao and Jin, 2020) and demonstrated that iPSCs-derived human neural progenitor cells (hNPCs), as well neurospheres, which is a model system of early neurogenesis and brain organoids, which represents a higher model of brain organization, can be infected by SARS-CoV-2 but not SARS-CoV.

As a common routine technique, multiple staining of the organoid allows one to follow the trafficking of the virus/viral proteins in the organelles. The organoids are fixed and stained with available antibodies (anti-SARS-CoV-2 spike, or Nucleocapsid antibodies, see **Table 3, Supplementary**). Alternatively, live imaging can be done with viruses tagged with GFP or mcherry for direct fluorescence. A last method used is the utilization of SARS-CoV-2-Pseudo-Entry Viruses, which are Recombinant VSV expressing SARS-CoV-2 spikes and detected by luciferase assay, especially for infectivity assays (Yang et al., 2020).

Fluorescence staining permits the measurement of the up or downregulation of host proteins, such as the ACE2 receptor and predict the infectivity rate of organs, as well as underline the importance of the co-receptors TMPRSS2 and TMPRSS4 in the infection (Zang et al., 2020). The virus or the viral proteins were co-localized with cellular markers such as neuronal, NPC markers (Zhang et al., 2020) or proliferating cells.

Once the model systems are established, they can be used for drug testing. Several drugs have proven their efficacy *in vitro*. Remdesivir inhibited SARS-CoV-2 replication in lung organoids (Pei et al., 2020), Imatinib, MPA, QNHC in lung and colonic organoids (Han et al., 2021), as well mycophenolic acid and quinacrine dihydrochloride (Han et al., 2021). Alternatively, neutralizing antibodies are a promising treatment modality in the fight of the virus as shown for antibody CB6 inhibiting viral replication in human airway and alveolar organoids (Han et al., 2021).

## High Content Imaging for Drug Screening

**Figure 10:**
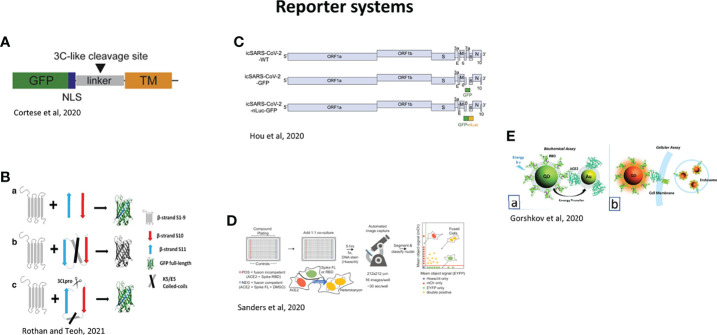
Reporter systems for the study of SARS-CoV-2. **(A)** GFP-NLS-tagged SARS-CoV-2 fluorescent reporter. NLS, nuclear localization sequence; GFP, green fluorescent protein; TM, transmembrane region of Sec61b. Black arrowhead: SARS-CoV-2 3C-like protease cleavage site. SARS-CoV-2-infected cells transiently expressing the GFP-NLS-tagged SARS-CoV-2 fluorescent reporter will show nuclear translocation of the GFP-NLS reporter (Cortese et al., 2020). **(B)** GFP-split complementation method. **(a)** When GFP is split into β1–9 and β10–11, GFP loses its fluorescence. β10–11 in anti-parallel position binds to β1–9, and green fluorescence is restored. **(b)** Insertion of E5/K5 heterodimer is used to flip β10 and β11 in parallel form and prevents self-assembly of the split GFP. **(c)** In the presence of the SARS-CoV-2, 3CLpro cleaves between E5/K5 heterodimer and β11, β11 flips back, which enables self-assembly with β1–9 and green fluorescence is restored (Rothan and Teoh, 2021). **(C)** Genomes of SARS-CoV-2 recombinant viruses. GFP or GFP-fused nLuc genes were introduced into the ORF7 (replacing aas 14–104) of SARS-CoV-2 genome (Hou et al., 2020). **(D)** Heterokaryon assay. Equal parts acceptor cells (express ACE2-iRFP and FUS-mCherry) plus donor cells (express spike FL-iRFP and HNRNPA1- EYFP) are co-cultured in 384-well microtiter plate: positive control (spike RBD, red column), negative control (DMSO, blue column), test compounds (other columns). After 5 hr, cells are fixed and nuclei are stained (Hoechst) then identified/segmented by automated confocal microscopy. Fraction cells fused is determined by percent co-positive (mCherry and EYFP) nuclei (Sanders et al., 2021). **(E)** Assays using nanoparticles. **(a)** Biochemical assay: the recombinant spike Receptor Biding Domain (RBD) is conjugated to fluorescent quantum dots (QDs), and ACE2 receptor is conjugated to gold nanoparticles (AuNP-ACE2). In case of binding of RBD to ACE2, energy transfer from Qd-RBD to AuNP-ACE2 occurs. **(b)** Cellular assay using QD-RBD interaction with ACE2 (with or without GFP modification at the end of the C-terminal) on the cell membrane (Gorshkov et al., 2020).

In difficult times like a pandemic, time can be saved with high throughput techniques which allow screening of drug compounds in large scale, in an automated manner. The easiest way is drug repurposing, where already approved drugs for other diseases are tested against new diseases, in this case COVID-19. As an example, inhibitors of coronaviruses have already been determined (Shen et al., 2019) and the libraries can be used now for SARS-CoV-2 (Ellinger et al., 2021). Moreover, high content screening (HCS) is also useful for discovery of new treatments and development of vaccines. Infection sensors in living cells were established to be able to indicate viral infection and test antiviral drugs. They have been used for HCS. GFP has been linked to a Nuclear Localization Signal (GFP-NLS) and a linker containing the cleavage site for SARS-CoV-2 main protease 3CL (Cortese et al., 2020; Pahmeier et al., 2021) (**Figure 10A**). This fusion protein is anchored in the cytosolic side of the ER by the transmembrane domain of Sec61β. If the cell is infected with SARS-CoV-2, 3CL cleaves the GFP-NLS, releasing the fusion protein, which then translocates to the nucleus. This system is adapted for live cell imaging and for screening of antiviral compounds. Co-expression of fluorescently labelled proteins of cytoskeleton with this sensor allows the study of intermediate filament rearrangement in infected cells (Cortese et al., 2020; Rothan and Teoh, 2021).

A HCS assay has been established using GFP-split complementation method to identify inhibitors of 3CLpro (Zhang et al., 2019; Rothan and Teoh, 2021) (**Figure 10B**). Basically, the assay consists of two plasmids; a first one with GFP and a second one with 3CLpro. GFP is itself splitted in two: GFP β1–9 and β10–11. In this configuration, GFP does not fluoresce. The cleavage site of 3CLpro is included in the expression cassette, and only after protease cleavage by 3CLpro, β10–11 binds to GFP β1–9, resulting in fluorescence. This system allows screening inhibitors of SARS-CoV-2 in biosafety level 2 (BSL2) without using the infectious virus. The study allowed the identification of several compounds including Boceprevir, Quinazoline, QZ1, QZ2, QZ3, and QZ5 (Rothan and Teoh, 2021).

Hou et al. (Hou et al., 2020) constructed 2 reporter viruses by replacing a 276 bp region in ORF7 by GFP or GFP-nanoluciferase genes (**Figure 10C**). They are useful to evaluate the SARS-CoV-2 pathogenicity and the efficacy of neutralizing antibodies.

A screening platform to evaluate syncytia formation by assessing heterokaryon formation, from 2 kinds of cells: donor cells expressing spike protein (green) and acceptor cells expressing ACE2 receptor (red) (**Figure 10D**) (Sanders et al., 2021). Other quantitative analyses in a virus-free system were realized to monitor cell entry by Zhang et al. with a fluorescent spike protein (Zhang et al., 2021d) (**Figure 10E**).

Some fluorescent based assays require Fluorescence Resonance Energy Transfer (FRET) to show viral infection (Brown et al., 2020; Zhu et al., 2020).

However, these experiments do not use microscopes, which could add additional information. As Zanella et al. stated, “seeing is believing” (Zanella et al., 2010). Hence the combination of high throughput techniques and cellular imaging has led to the development of a multidimensional technique, which allows for high content screening (HCS). Multiple quantitative and qualitative results can be obtained, leading to a complete analysis, including number of fluorescent cells, measurement of fluorescence intensity, subcellular localization, cell morphology and cell movement and geographical cell distribution. HCS is much more informative than conventional flow cytometry. Although the system does not reflect the *in vivo* conditions, it is adequate for the initial stages of drug screening. It can predict promising drug candidates, by testing first their cytotoxicity, solubility, specificity and absorption, distribution, metabolism and excretion (ADME) properties at a single cell level, optimization before *in vivo* studies. HCS used to be based on fixed cells but it has been widely improved with live cell imaging, 3D models (Kunz-Schughart et al., 2004) and the culture of primary human cells (Evensen et al., 2010).

Few studies have used microscopy to test the efficacy of drugs. Many host-targeting antivirals, previously used for other CoVs infections, have been tested for SARS-CoV-2 (Mei and Tan, 2021; Zhou et al., 2022) but only a few used microscopy techniques (**Table 2**). Some antivirals can target the host machinery by blocking the binding of ACE2 and RBD, inhibiting endocytosis, playing a role in immunoregulation and cellular pathways, inducing or inhibiting autophagy, or acting as protease inhibitors. Few studies have been done on SARS-CoV-2 infected cells (Wang et al., 2020b).

**Table 2:** New therapeutic targets against SARSCoV2 showing manuscripts that used microscopy experiments for testing drugs for SARS-CoV-2 inhibition.

Inhibitor/drug	Target	Articles	Effect
Naringenin	Endo-lysosomal Two-Pore Channels (TPCs)	(Clementi et al., 2021)	Inhibition of SARSCov2 infection
Camostat mesylate (TMPRSS2 inhibitor) Nafamostat mesylate (TMPRSS2 inhibitor) CA-074 methyl ester (CTSB inhibitor) Z-Phe-Tyr(tBu)-diazomethylketone (CTSL inhibitor)	TMPRSS2 (membrane protein) CTSB (cathepsin B, lysosome), CTSL (cathepsin L, lysosome),	(Hashimoto et al., 2021)	Inhibition of SARSCov2 infection
**PPMO T-ex5** Inhibitors of TMPRSS2: Aprotinin, MI-432, and MI- 1900) Furin inhibitor (MI-1851) Endosomal cathepsins inhibitor (E64d)	TMPRSS2 (Transmembrane serine protease 2) Furin Endosomal cathepsins	(Bestle et al., 2020)	MI-1851 inhibits SARSCoV2 replication E64d did not affect virus replication
Inhibitors of androgen receptor (AR) Bromodomain and extraterminal domain (BET) inhibitor	TMPRSS2 (transcriptional regulation) ACE2 Androgen receptors	(Qiao et al., 2020)	Prevents SARSCoV2 infection
**Controversial:** **Chloroquine (CQ)/Hydroxychloroquine (HCQ)**	pH inhibition of endosomal acidification	(Lv et al., 2021) (Wang et al., 2020b)	Increase sarscov2 infection by macrophage polarization (increase of endosomal pH). Inhibition of infection Inhibition of infection
**Anti-vimentin antibody**	**Vimentin**	(Cortese et al., 2020)	Blocks viral replication

Morphological profiling and machine learning for rapid diagnosis are also essential tools in HCS technology. A high content microscope associated to spinning disk was used (Mirabelli et al., 2020) to capture cell events related to viral infection such as cell morphology, cytoskeletal or cytoplasmic characteristics. However, it can also be captured with simple fluorescent microscope associated to HCS. After manual validation with infected and non-infected cells, the machine learns to recognize characteristics of infected cell. Morphological profiling was then used to recognize cell characteristics associated to SARS-CoV-2 infection, which are formation of syncytia, increased nucleoli count, cytoplasmic protrusions, rearrangement of host proteins, or detection of viral events such as viral entry or viral replication. Once machine learning is set up, a panel of drugs is assessed to test their antiviral activity. With this technique, 17 FDA-approved drugs have been identified to decrease SARS-CoV-2 infection (Mirabelli et al., 2020).

## High-End Technologies With Potential to be Used in the Study of SARS-CoV-2

Priority now is to develop vaccines and therapeutics against SARS-CoV-2. Various bioengineering tools developed for virology and pharmacology are already available and can be used (Raimondi et al., 2020) for pre-clinical testing, which can save time. Importantly, most of these tools only require an optical microscope and can be used widely, especially in developing countries.

In scaffolds, biomaterials are used to form a structure where cells are displayed. In bioprinting systems, cells are distributed in a hydrogel system, allowing thinner and more homogeneous cell layers, required for a better surface exchange like the air-blood tissue barrier engineered (Horvath et al., 2015). Bioinks have already been used for the study of influenza virus (Berg et al., 2018). Microfluids additionally mimic air and fluid flow. Especially, the organ-on-a-chip technology, which can be lung/kidney/heat/multi-organs-on-a-chip refers to an organ mimetic on a microfluidic chip (Wu et al., 2020c). Huh et al. have described an alveolar-capillary interface of the human lung, which could be a model for SARS-CoV-2 (Huh et al., 2010). The limitation with scaffolds made with hydrogel or solid ECM is that they are often very thick and imaging with optical microscope is impossible. Some groups have developed mini scaffolds, such as Nichoid (Raimondi et al., 2012), which is a transparent grid, up to 100 microns and allowing high resolution fluorescent imaging for diagnostics. A millifluidic optically-accessible bioreactor (MOAB) has also been developed (Lagana and Raimondi, 2012). This microfluidic bioreactor is a 400 μm thick chambered microscope slide, containing millions of cells infused with the drug to be tested and allowing high resolution imaging especially in real time. An upgrade of HCS for drug testing consists on using micro scaffold array chip (Li et al., 2014) allowing 3D culture, drug administration and quantitative analysis. A device with microfluidic system (Lim and Park, 2018) has even been built allowing the growth of spheroids with a concentration gradient of drugs, which mimics the *in vivo* conditions. Different models do exist (Huh, 2015; Knowlton and Tasoglu, 2016; Miccoli et al., 2018) and could be used in the fight of COVID-19.

Intravital imaging models are another option for the fight against COVID-19. A miniature device called MicroAtlas (Raimondi et al., 2020) is composed of a fluorescent 3D scaffold, adapted for deep optical sectioning with a two-photon microscope. With this device, optical aberrations are eliminated, the field of view of the microscope is corrected. Minimally invasive compared to the usual devices, it allows quantitative analysis of host response or monitoring drug release or testing vaccines *in vivo*, without sacrificing an animal and then allowing the measurements at different time points. As an example, SARS-CoV-2 RNA vaccines can be tested by fluorescently labelling the mRNA used and following its localization in the cells and its translation into the viral protein. This could allay fears of the persistence of genomic material in the cells and alterations of the human genome.

Additionally, some techniques such as Raman microscopy, especially non-linear coherent anti-Stokes Raman scattering microscopy (CARS) (Tabish et al., 2020) or tip-enhanced Raman spectroscopy (TERS) (Deckert et al., 2020) are promising techniques and could be developed for rapid viral detection in patient samples. CARS measures the molecular vibrations of a molecule and allows a 3D representation of molecular vibrations. CARS is able to detect SARS-CoV-2 without any labelling, and then limits photodamage usually due to fluorescence. Compared to conventional microscopy, CARS also has a high penetration depth (several hundred micrometers), high spatio-temporal resolution [lateral resolution of 0.3 μm and depth resolution of 0.9 μm (Cheng, 2007)], and high acquisition speed. TERS combines atomic force microscopy (AFM) or scanning tunnelling microscopy (STM), which resolve morphological details, and Raman microscopy, which provides spectral information of the sample surface. As a result, a spatial resolution of 0.5-1nm can be reached, allowing detection of viral components such as nucleic acid or glycoproteins. TERS has already been used to differentiate viral strains of differing sizes (Hermann et al., 2011) and could constitute a powerful tool for rapid detection of SARS-CoV-2 infection in tissues.

These techniques, in combination with large scale screening, are the future of drug discovery and are essential for pre-clinical studies, not only for COVID-19 but also for future pandemics. They could be used for precision medicine too as reactions to the virus are different for each person, depending on their genetic background or immunity.

## Conclusion and Future Recommendations

Microscopy, in all its forms, from classical to currently developed novel methods have played a pivotal role in our understanding the SARS-CoV-2, its pathogenesis and evasion of the immune response. Microscopy has the huge advantage in providing different facets of information in one slide such as detection of the virus, localization, multiple labelling and quantification compared to RT-PCR, standard method, which can only inform on the presence/absence of the virus. Microscopy techniques have however some limitations, such as the sensitivity and specificity of IHC/ISH, or the difficult diagnosis by EM, but these limitations can be overcome by association of different techniques to indisputably give a conclusion on the presence of the virus, such as the correlative light-electron microscopy, or a complementation of light microscopy results by RT-PCR. Of course, microscopy results are valid only when accompanied with the right positive and negative controls. One has to be careful when analysing the results, many articles have been withdrawn following recognition of the false identification of the virus. **Table 3, Supplementary** lists retracted papers, which used microscopy techniques (Retraction Watch). New *in vitro* models such as organoids, which give a better representation of the *in vivo* situation, combined to high resolution techniques such as FIB-SEM, LSFM, electron tomography and PC-CT, allow the 3D reconstruction of the infected cells and are very useful to understand the viral multiplication cycle. They also showed a complete hijacking of the cell by SARS-CoV-2 at different levels, such as the autophagy pathway, an entire reorganization of the cytoskeleton and the organelles. We highlighted the interference of the virus with immunity leading to the “cytokine storm”, which can induce injuries in a high variety of organs and can lead to death, or participate to age-related diseases. High scale, rapid and sensitive tests have been developed as they are essential in a pandemic for the detection of the infection and the use of artificial intelligence and deep learning. High content screening is also used for drug testing, several drugs have been identified as infection inhibitors.

Despite its devastating consequences, the COVID-19 pandemic has accelerated the development of novel techniques and reagents in order to study SARS-CoV-2. All these advances are not exclusive to understanding SARS-CoV-2 but have contributed to science in general, particularly in the field of virology. However, numerous promising techniques still have to be explored, to generate the knowledge necessary to prevent and treat this infection that has changed our world irrevocably.

## Author Contributions

AD carried out the literature review, wrote the first draft and prepared all figures and tables. AK conceptualized the research, revised and edited the manuscript. All authors contributed to the article and approved the submitted version.

## Conflict of Interest

AK is an associate editor of Frontiers in Microbiology.

The remaining author declares that the research was conducted in the absence of any commercial or financial relationships that could be construed as a potential conflict of interest.

## Publisher’s Note

All claims expressed in this article are solely those of the authors and do not necessarily represent those of their affiliated organizations, or those of the publisher, the editors and the reviewers. Any product that may be evaluated in this article, or claim that may be made by its manufacturer, is not guaranteed or endorsed by the publisher.
