# Supplementary material for: Imaging Techniques: Essential Tools for the Study of SARS-CoV-2 Infection
Source: Front Cell Infect Microbiol. 2022 Jul 22;12:794264. doi: 10.3389/fcimb.2022.794264 (PMC9355083; doi:10.3389/fcimb.2022.794264)
Supplement: Supplementary file 1 [file Table_1.docx]

**Table 1**: **A.** List of microscopy techniques used or with a good potential for the study of SARS-CoV-2. Principle, advantages and limitations are described. The differences between the light, electron, and correlated microscopy are mentioned. Details of different microscopes belonging to either light or electron microscopy are listed. **B.** List of microscopy staining techniques used for the study of SARS-CoV-2, with their principle, advantages and limitations.

| 1. **Microscopy techniques** | **Principle- advantages** | **Limitations** |
| --- | --- | --- |
| **Light microscopy (LM)** | *Principle:* uses visible light and a system of lenses to detect and magnify small objects.  *Resolution:* ~200nm laterally, 500nm axially  *Advantages:*   - Several probes/colours can be used simultaneously. - Locates rare events - Labels specific components - No coating needed. Gives information about the structure. - Live cell imaging is possible (dynamic information) - Understand the relationship between the cells, highlight organelles. Look at only fluorescent objects. - Lower sample damages. | - Dyes may impact on the cell physiology/ protein function - Autofluorescence - Photobleaching - labelling is time consuming - phototoxicity |
| **Electron microscopy (EM)** | *Principle:* A beam of electrons goes through the specimen. Signals coming from the interaction between the sample and the electron beam give informations about structure, morphology and composition.  *Resolution:* up to 0.2nm (1000X more than for light microscopy)  *Advantages:*   - High magnification. | - No live cell imaging. Dry samples in a vacuum. - Heavy preparation, which could modify the native state. Sample damages. Special sample preparation: ultrastructure has to be preserved, heavy metals may be needed for higher contrast, sample has to be stable in the vacuum. - Possible artefacts - Costs (although costs can be similar to a confocal microscope). - Black and white images (false colours can be added) - Size of the equipment - Training can take years - Sample has to be thin enough for electron beam penetration (500nm). - Gives a 2D projection of small cell volume (50-500nm thick, some microns wide). To be able to do 3D imaging, serial sections are needed. - Difficulties to find rare events. |
| **Correlative light and electron microscopy (CLEM)** | *Principle*: to complement static EM image with dynamic live imaging.  *Advantages:*   - Combine the advantages of fluorescent imaging (spatial and temporal) to the high resolution of EM. Overcomes limitations of light microscopy/ EM used alone. - Locate virions at the surface of the cell with EM (and determine their morphology) and add dynamic data from fluorescent staining. Molecular composition, protein distribution are available. Can find rare events.   LM can be incorporated at any stage: 3 different techniques  -LM before embedding: uses standard EM and LM protocols  -LM after embedding: the same state of the specimen is imaged with LM and EM.  -cryo-LM (with no embedding): fluorescence is preserved with cryo conditions. | - Antibody penetration - Limited number of cells - Limited number of wavelengths (number of laser lines) available.   -LM before embedding: the structures may change between LM and EM due to movements inside the cell or due to sample preparation.  -LM after embedding: autofluorescence of the resin, loss of fluorescence.  -cryo-LM (with no embedding): needs cryo LM setups (to maintain samples at low temperature). |
| **Microscope** | **Features - resolution** | **Limitations** |
| **Fluorescent light microscope** | *Principle:* optical microscope which uses fluorescence to visualize events. Protein of interests are tagged with a fluorophore or immunostained. In this case, antibodies are coupled to a fluorophore. The fluorophore is excited with a wavelength and reemits light at a higher wavelength.    *Optical resolution in z*: 200-300nm  *Advantages:*   - Simple to use, low cost | - Illuminates all the sample: photons coming from out-of-focus regions resulting in a high background signal. - Limited resolution - No 3D |
| **Confocal microscope** | *Principle* : Presence of a pinhole in front of the detector: Images from the focal plane, the light coming from above and below the focal plane is eliminated.  Improvement in axial resolution compared to widefield (180nm laterally, 500nm axially)  *Advantages:*   - Background is eliminated compared to fluorescent light microscope, due to the pinhole. - Beam of light, only a single point is illuminated at a time. - Quantitative tool - Like a flow cytometer but allowing correlation between cellular location and signal intensity. - Z-stacks can be done. - Multispectral analysis (through the visible range light) - Increased SNR compared to widefield. - Numerous applications: protein trafficking, co-localization, 3D, optical sectioning. - Live cell imaging can be done (time-lapse: 4D imaging) - Improvement of the accuracy for the colocalization studies compared to fluorescent light microscope. | - Still diffraction-limited - Can only penetrate sample up to 50µm due to light scattering. - Compromise between resolution, speed and sensitivity (a higher resolution needs longer scanning but the photodamage is more important). - Phototoxicity and photobleaching (because of oversampling) due to high laser power. |
| **Airyscan** | *Principle*: Array detector of 32 elements of 0.2 Airy units, each one acting as a small pinhole.  *Resolution:* “near to super-resolution”. 120nm lateral and 350nm axial.  *Advantages:*   - Add-on on a confocal microscope - Improvement in resolution, increased by a factor of 1.7 in all spatial directions compared to confocal; more photons are collected, increasing signal to noise ratio (SNR), sensitivity, speed or resolution. - Same sample preparation than for confocal. - Increase accuracy of colocalization studies compared to confocal imaging. | - Compromise between speed, sensitivity and resolution. - Phototoxicity and photobleaching due to high laser power. |
| **Stimulated emission depletion (STED)** | *Principle:* 2 laser beams: a laser pulse and a doughnut-shape depletion laser beam. Fluorescence is depleted in some areas of the sample, and the central focal spot remains active. This decreases the area of illumination at the focal point.  *Resolution*: <20 nm lateral and 40-50nm axial. (super-resolution microscopy)  *Advantages:*   - Diffraction limit is overcome - No need for computational reconstruction - Fast scanning, good penetration depth, multicolour - No need of specialized fluorophores | - Complex optical set-up - Photobleaching and phototoxicity due to high laser power. - Little improvement in z-resolution compared to confocal. |
| **Light Sheet Fluorescent Microscopy** **(LSFM)**  **(Selective Plane Illumination Microscopy (SPIM))** | *Principle*: orthogonal light sheet excites a small region in the focal plane. Images are captured from different views, 3D image is reconstituted. Good for thick samples.  *Resolution:* comparable to epifluorescent microscopy- 250-500nm. Axial resolution even worse.  *Advantages:*   - Samples scanned with a plane of light= increased speed of acquisition (10-100 times compared to confocal), increased SNR. - Good optical sectioning. This reduces the background, leading to high contrast images comparable to the confocal. - Visualize more 3D/4D samples than traditional fluorescent microscopy. - Reduces the photodamages and stress in living samples compared to other microscopy techniques (photobleaching reduced 100 times compared to confocal). | - Relatively low resolution |
| **Inverted Selective Plane Illumination Microscopy (iSPIM)** | *Principle:* Extension of LSFM for single view sample preparation with 2 perpendicular objectives, placed at right angle above the sample, at 45 degrees from vertical; 1 objective creates the light sheet, the second one collects the stack of images. Uses a regular inverted microscope. Non-invasive high-speed volumetric imaging of living samples.  *Resolution:* ~500nm laterally and 170nm axially.  *Advantages:*   - Add-on on an inverted microscope - Possibility to do live cell imaging. - monolayers of cells on a coverslip or cells in a microfluidic device - 30 times faster than spinning-disk confocal microscopy, with a comparable SNR. The high speed reduces the motion blur seen in live cell imaging. - Reduces phototoxicity | - Loss of working distance |
| **Dual inverted Selective Plane Illumination (diSPIM)** | *Principle:* Variation of LSFM. 2 excitation sheets are used. The same 2 perpendicular objectives from iSPIM are used but the role of the objectives is reversed. 2 datasets of images are created. Contains 2 scanners and 2 cameras.  *Resolution:* 330nm in x, y and z.  *Advantages:*   - Can be mounted on an inverted microscope. Very flexible light-sheet microscope. - Like other SPIM, it illuminates only the focal plane and minimizes phototoxicity (photobleaching is reduced >10 times) - ~2 times improvement in axial resolution, speed comparable to spinning disk confocal microscopy. - Simple sample mounting compared to other SPIM. - Improved illumination homogeneity, larger sample sizes, increased axial resolution, and enhanced signal to noise response, decreased photobleaching. - 3D reconstruction - High speed: 200 images/s | - The 2 views have to give same image quality, the object does not have to move, otherwise the image reconstruction leads to artifacts - Huge dataset |
| **Propagation-based X-Ray phase contrast tomography (PB-CT)** | *Principle*: 3D X-ray imaging that uses refraction and absorption of x-rays in the tissues to increase the SNR compared to conventional absorption-only x-ray tomography.  *Resolution*: at a few µm, isotropic resolution (equal pixel size in x, y and z). Subcellular resolution, thick tissues.  *Advantages:*   - 3D reconstruction with no destruction of the specimen compared to conventional histology. - Selectable fields of view, in the range of several mm, and voxel sizes in the range of several µm. |  |
| **Scanning electron microscope (SEM)** | *Principle*: Scanning of the sample by high energy electron beam, detection of the surface with scattered electrons*.*  *Resolution:* ~1nm.  *Advantages:*   - 3D-like and topographical information - Great depth of focus - Max magnification: 500 000 times. - Easy to operate, fast acquisition - Minimal preparation | - Special training for limitations of artifacts in sample preparation - Samples must be solid and vacuum-compatible - Samples must be coated with gold or carbon |
| **Cryo electron microscope (Cryo-EM)** | *Principle:* For delicate specimen that contains liquid. Scanning of a thin layer of frozen sample.  *Advantages:*   - No artifacts due to fixation/dehydration - Preservation of native conformation - No stain is needed | - Low SNR due to ice thickness - Freezing artefacts |
| **Transmission electron microscope** **(TEM)** | *Principle:* The electron beam goes through a thin sample, and the transmitted electrons are detected.  *Resolution:* ~200-0.2nm  *Advantages:*   - Used to view thin sections of cells, structure of molecules, organization of viral proteins. - Max magnification: 5 000 000 times | - Only 2 dimensions - Samples subject to electron bombardments - Lots of sample preparation - Cannot be used in live samples. |
| **Focused Ion-beam – Scanning Electron microscope (FIB-SEM)** | *Principle*: High resolution 3D SEM analysis in x,y,z. The beam of electrons of SEM is replaced by a focused beam of ions. Also called volume EM (vEM), large-scale volumetric imaging.  *-Resolution* *x*,*y,* z ~ 10 nm  *Advantages:*   - 3D resolution over thousands of cubic microns (sacrifice of some resolution compared to TEM but gain of a 3^rd^ dimension) - Automated acquisition - Little artifacts - Captures virus-cell interactions. Quantitation of membrane curvatures and viral densities. Applicable on samples embedded for TEM. | - No post-staining of sample - Not a high-throughput approach: ~10,000 µm3 or a volume of 25 × 20 × 20 µm per day, without compromise of resolution or SNR - Expensive consumable |
| **Cryo-FIB-SEM** | *Principle*: FIB-SEM at cryo conditions, in the absence of heavy metal staining/embedding.  *Resolution* : same than a SEM (~1-2nm).  *Advantages:*   - Fast 3D cryo-imaging of large native frozen tissues. - Whole cell imaging of unstained samples. | - Some samples can still be sensitive to the process - Some cellular components are still not well identified |
| **Electron tomography** | Principle: Volume EM, high TEM resolution in x, y and z  *Resolution*: x,y,z~ 2nm | - limited to small volumes (z ~ 300nm) |
| **Raman** | *Principle*: scattered light is used to measure the vibrational energy modes of a sample.  *Resolution:* 1µm-400nm, but can approach diffraction limit.  *Advantages:*   - no sample preparation - label-free - non-destructive - highly specific - acquisition in few seconds | - Fluorescence of impurities can hide the Raman spectrum - Laser can destroy the sample - Several hours of acquisition |
| **Coherent Anti-Stokes Raman Scattering (CARS)** | *Principle*: dye-free imaging based on the vibrational contrast of the molecules in the sample.  *Resolution*: 328nm  *Advantages:*   - As dye-free, the sample is unaffected. - Multiple molecules can be visualized in the same time - Reduced time of acquisition compared to Raman (several minutes) - Deep penetration of the tissues - High sensitivity - Chemical specificity - 3D resolution - Reduced photodamage | - Different technical barriers prevent its development in clinical applications:   -e.g, collection of the signal at one Raman frequency, different molecules cannot be differentiated.  -Difficulty to differentiate the signal from the background |
| **Tip-enhanced Raman spectroscopy (TERS)** | *Principle*: Specialist approach to [surface-enhanced Raman spectroscopy](https://en.wikipedia.org/wiki/Surface-enhanced_Raman_spectroscopy), with enhancement of Raman scattering, with gold coating. Requires a confocal microscope and a scanning probe microscope.  *Resolution*: spatial resolution 8nm.  *Advantages:*  Enhancement of resolution compared to normal Raman. |  |
| **Scanning tunnelling microscope (STM)** | *Principle*: images surfaces at the atomic level, using a sharp tip sensing the surface of the sample.  *Resolution*: 0.01nm  Advantages:   - Can be use in vacuum, air, liquid environment - Provide a 3D profile of the surface - Determination of the size and conformation of molecules at the surface | - Can be difficult to use - Requires very stable and clean surface, good vibration control and sharp tips. - Fragile and expensive equipment. |

| 1. **Staining techniques** | **Principle** |
| --- | --- |
| **Haematoxylin and eosin (H&E)** | Standard tissue stain used in histology/histopathology. Hematoxylin has a deep blue-purple colour and stains nucleic acids (chromatin, ribosomes, cytoplasmic RNA). Eosin is pink and stains proteins. Nuclei are stained in blue, and cytoplasm and extracellular matrix have different degrees of pink. *Advantage*: Does not depend on fixation technique.  *Limitation*: Contrast is not always optimum. |
| **Immunohistochemistry (IHC)** | Immunostaining: it involves antibodies which recognize the antigens. It can be direct (with only the primary antibody) or indirect (with primary and secondary antibody).  Visualization can be :  -**chromogenic**, where the antibody is linked to an enzyme such as peroxidase. *Advantages:* greater sensitivity compared to IF, long lasting signal. *Limitations*: difficult for colocalization, low dynamic range, difficult multiplexing.  e.g: 3’diaminobenzidine (DAB): a primary antibody binds to the target protein, a biotin-tagged secondary antibody bids to the primary, a streptavidin-peroxidase complex binds to the biotin, and produces a dark brown reaction product.  -with **immunofluorescence** (IF), where the antibody is linked to a fluorophore. *Advantages*: fewer steps, multiplexing, colocalization studies, high dynamic range (detection of rare and high abundant targets). *Limitations*: photobleaching, lower sensitivity compared to chromogen. |
| **Negative staining** | Sample is unstained, background is stained; the specimen is embedded in electron-dense metal atoms, the light goes through the specimen but not through the metallic background, giving a bright specimen on a dark background.  Determination of morphology and subcellular components, as well macromolecules (DNA and proteins).  *Advantages:* single stain, quick technique; does not need heat-fixing so the cells are not deformed; cells are easy to be localized. *Limitations:* does not provide a lot of information about the cell; specific strains/types of organism cannot be differentiated. |
