# Supplementary material for: Imaging Techniques: Essential Tools for the Study of SARS-CoV-2 Infection
Source: Front Cell Infect Microbiol. 2022 Jul 22;12:794264. doi: 10.3389/fcimb.2022.794264 (PMC9355083; doi:10.3389/fcimb.2022.794264)
Supplement: Supplementary file 3 [file Table_3.docx]

# Table 3: Retracted papers which included microscopy and artificial intelligence (from Retraction Watch, Retracted coronavirus (COVID-19) paper, https://retractionwatch.com/retracted-coronavirus-covid-19-papers/).

| **Title** | **Journal** | **Date of publication** | **Date of retraction** |
| --- | --- | --- | --- |
| A deep learning model and machine learning methods for the classification of potential coronavirus treatments on a single human cell | Journal of Nanoparticle Research | October 17, 2020 | August 16, 2021. |
| Acute kidney injury and collapsing glomerulopathy associated with COVID-19 and APOL1 high risk genotype,” Abstract 111 and Abstract 621 | Journal of Investigative Medicine | - | April 1, 2021 |
| AI Techniques for COVID-19 | IEEE | July 8, 2020 | ? |
| Autopsy and Histologic Findings of Patients with New Coronavirus Pneumonia: The Pathologic Associations with Hypoxemia | Medical Science Monitor | February 13, 2020 | March 17, 2021. |
| System to screen coronavirus disease 2019 pneumonia | Applied Intelligence | April 22, 2020 | ? |
| Detecting Subacute Thyroiditis after COVID-19 infection using Deep Learning Techniques | Journal of Physics: Conference Series | April 23, 2021 | December 21, 2021. |
| SARS-CoV-2 infects T lymphocytes through its spike protein-mediated membrane fusion | Cellular & Molecular Immunology | April 7, 2020 | July 10, 2020. |
